# Supplementary material for: Towards spatially selective efferent neuromodulation: anatomical and functional organization of cardiac fibres in the porcine cervical vagus nerve
Source: J Physiol. 2024 Aug 26;603(7):1983–2004. doi: 10.1113/JP286494 (PMC11955868; doi:10.1113/JP286494)
Supplement: Supplementary file 2 — Supplementary Information [file TJP-603-1983-s002.pdf]

Supplementary Information

Towards spatially selective efferent neuromodulation: Anatomical and functional organization of cardiac fibers in the porcine cervical vagus nerve

Nicole Thompson, PhD,*[a] Enrico Ravagli, PhD,*[a] Svetlana Mastitskaya, PhD,[a] Ronald Challita, MD,[b] Joseph Hadaya, MD, PhD,[b] Francesco Iacoviello, PhD,[c] Ahmad Shah Idil, BEng,[a] Paul R. Shearing, PhD,[c] Olujimi A. Ajijola, MD, PhD,[b] Jeffrey L. Ardell, PhD,[b] Kalyanam Shivkumar, MD, PhD,[b] David Holder, PhD,[a] Kirill Aristovich, PhD[a]

*Joint first authors

[a] EIT and Neurophysiology Research Group, Department of Medical Physics and Biomedical Engineering, University College London, London, United Kingdom

[b] UCLA Cardiac Arrhythmia Center and Neurocardiology Research Program of Excellence, David Geffen School of Medicine at UCLA, Los Angeles, California, USA

[c] Electrochemical Innovation Lab, Department of Chemical Engineering, University College London, London, United Kingdom

Section 1

Supplementary Table 1.1: Coordinates of electrodes over the cross-section

Pair	**1**	**2**	**3**	**4**	**5**	**6**	**7**	**8**	**9**	**10**	**11**	**12**	**13**	**14**
Electrode X (um)	-337	-944	-1348	-1500	-1348	-944	-337	337	944	1348	1500	1348	944	337
Electrode Y (um)	1462	1166	658	0	-658	-1166	-1462	-1462	-1166	-658	0	658	1166	1462

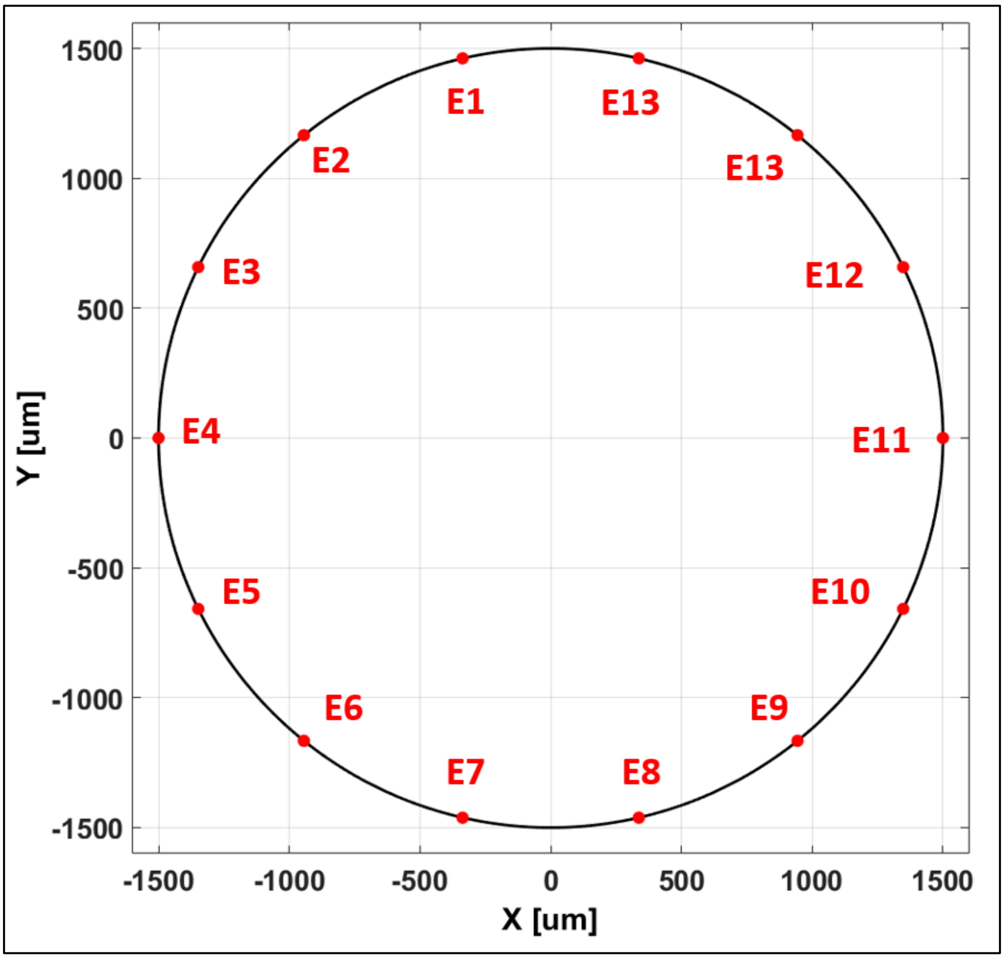

Supplementary Figure 1.1: Coordinates of electrodes (E) over the cross-section

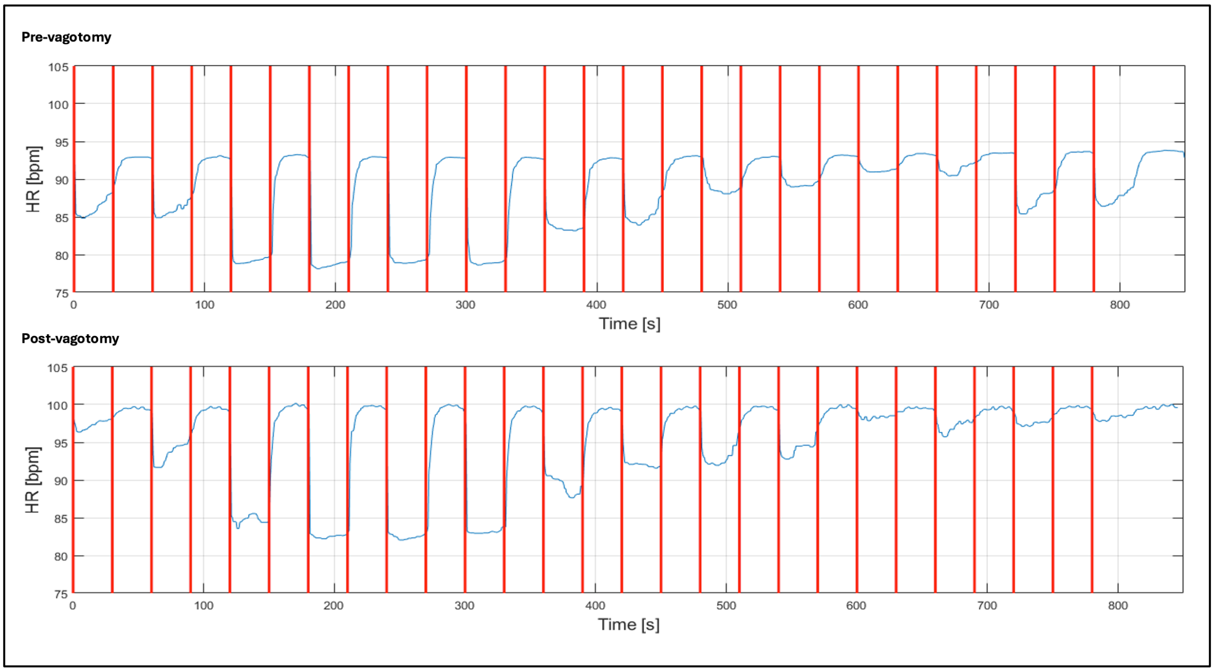

Supplementary Figure 1.2: Cardiac effect from activation of the right vagus nerve pre- and post- right proximal vagotomy. As a control to determine any influence or effect of the afferent fibres on the right vagus nerve efferent (bradycardia) effect when stimulated with parameters at a lower threshold (1mA, 1ms) than required to activate smaller, afferent fibres, the cardiac effect was measured before and after a proximal right vagotomy. The percent of change on each electrode was similar between the two measurements, with $P<0.05$. The first and last few electrodes showed a slight variation in response; however, this is due to the shunting of the electrodes at the opening of the cuff due to flooding with fluid. Both this and the difference in the baseline heart rate can be explained by the length of time between the measurements during the experiment, of approximately three hours, due to multiple other recordings taking place before the vagus nerve could be cut for this control. HR = heart rate, bpm = beats per minute, s = seconds, red bars = stimulation on/off alternating over time, across 14 electrode pairs.

Section 2
Raw data for electrophysiology/selective nerve stimulation (sVNS):

Supplementary Table 2.1: Cardiac Pre-vagotomy sVNS Data

Animal Number	Vagus	Filename in Dataset	Pulse Features		Heart Rate [bpm] - Stimulation Pair vs Off-Time																											
			Amplitude [µA]	Width [µs]	1	Off	2	Off	3	Off	4	Off	5	Off	6	Off	7	Off	8	Off	9	Off	10	Off	11	Off	12	Off	13	Off	14	Off
1	Right	UCLA_2021_Pig2_RV_0p8mA_1ms_30on_30off_20Hz	800	1000	95.8	108.9	96.4	108.4	98.4	108.2	101.1	108.3	108.5	108.9	109.2	109.2	109.3	109.3	109.6	109.7	109.6	109.6	109.7	109.6	109.7	109.6	99.6	109.6	97.9	108.8	109.0	108.9
2	Right	UCLA_2021_Pig3_RV_1mA_100us_30on_30off_20Hz_fullcircle	1000	100	71.2	78.5	65.8	78.2	71.4	77.9	77.1	78.0	77.9	78.2	78.2	78.4	78.5	78.6	78.6	78.6	78.6	78.8	77.7	78.7	78.4	78.6	78.4	78.7	78.4	78.7	78.5	78.7
3	Right	UCLA_2021_Pig4_RV_1mA_0p1ms_30on_30off_20Hz	1000	100	106.0	106.2	105.6	106.4	105.6	106.1	104.8	106.1	104.0	106.1	102.9	105.7	103.4	105.9	103.8	106.2	106.1	106.3	106.2	106.3	106.3	106.3	106.4	106.4	106.3	106.5	106.9	106.9
4	Right	UCLA_2021_Pig5_RV_SS_2mA_4ms_20Hz_30onoff	2000	4000	70.5	74.6	69.4	74.2	74.3	74.6	74.6	74.9	74.2	75.2	75.0	75.2	75.1	75.3	74.9	75.3	74.8	75.2	74.7	75.2	75.4	75.5	69.7	74.8	68.0	74.4	73.9	74.7
5	Right	UCLA_2021_Pig6_RV_SS_1mA_1ms_20Hz_30onoff	1000	1000	114.0	113.9	113.2	113.8	108.0	113.1	109.1	113.0	110.9	113.0	113.3	113.5	113.6	113.6	113.8	113.8	113.8	113.9	113.9	114.1	114.1	114.0	114.0	114.0	114.0	113.9	113.8	113.7
6	Right	UCLA_2022_Pig2_20_SS_RV_Cardiac_0p5mA_1ms	500	1000	61.7	66.7	61.1	67.0	58.8	67.2	67.5	66.5	66.4	66.2	66.6	66.1	66.7	66.3	66.2	66.2	66.7	66.4	66.8	66.3	66.9	66.2	66.6	66.6	61.2	67.2	61.0	66.7
7	Right	UCLA_2022_Pig3_18_sVNS_RV_Cardiac_2mA_4mS	2000	4000	103.2	103.5	102.6	103.3	103.1	103.1	102.4	103.2	102.7	103.4	102.7	103.6	102.6	103.6	102.9	103.8	102.7	104.0	102.0	104.2	95.5	104.5	102.3	103.7	102.5	103.5	103.3	103.1
8	Right	UCLA_2022_Pig4_17_SS_RV_Cardiac_2mA_4ms	2000	4000	86.6	90.0	87.7	90.1	89.2	90.4	90.2	90.8	90.3	91.0	90.5	91.0	90.6	91.3	90.8	91.3	90.0	91.1	83.5	90.3	81.5	89.8	86.3	89.9	89.7	90.3	90.6	90.8
9	Right	UCLA_2022_Pig5_27_SS_RV_Cardiac_1mA_1ms	1000	1000	62.6	66.4	64.4	66.4	65.5	66.5	64.7	66.6	63.1	66.5	66.9	66.8	66.2	66.9	60.9	66.5	60.6	66.0	60.5	65.9	60.8	66.0	59.5	66.2	64.8	66.5	65.3	66.6
10	Right	UCLA_2022_Pig6_30_SS_RV_1mA_1ms_new_cuff	1000	1000	68.2	68.1	66.5	68.1	68.2	68.1	68.0	68.4	68.1	68.3	67.9	68.5	68.1	68.5	68.1	68.5	68.2	68.3	67.8	68.2	68.1	68.1	68.0	68.0	62.3	68.0	68.0	68.4

Supplementary Table 2.2: Cardiac Pre-vagotomy sVNS Data

Animal Number	Vagus	Filename in Dataset	Pulse Features		Heart Rate [bpm] - Stimulation Pair vs Off-Time																											
			Amplitude [µA]	Width [µs]	1	Off	2	Off	3	Off	4	Off	5	Off	6	Off	7	Off	8	Off	9	Off	10	Off	11	Off	12	Off	13	Off	14	Off
1	Right	UCLA_2021_Pig2_RV_2mA_20ms_30on_30off_20Hz_Right_distal_vagotomy	2000	2000	107.00	107.00	107.00	107.00	107.00	107.00	107.00	107.00	106.50	107.00	106.00	106.90	105.00	106.50	104.50	106.50	105.00	106.50	106.00	106.50	105.50	106.50	106.50	106.50	106.50	106.50	106.50	106.50
2	Right	UCLA_2021_Pig3_RV_2mA_40ms_30on_30off_20Hz_Right_distal_vagotomy	2000	4000	73.0	73.0	73.0	73.0	72.5	73.0	71.0	73.0	71.0	73.0	71.5	73.0	72.0	73.0	73.0	73.0	73.0	73.0	73.0	73.0	73.0	73.0	73.0	73.0	73.0	73.0	73.0	73.0
3	Right	UCLA_2021_Pig4_RV_2mA_4ms_30on_30off_20Hz_postVagotomy_postEIT_rep1_evenPairs	2000	4000	113.00	105.00	113.00	105.00	105.00	105.00	105.00	105.00	105.00	105.00	105.00	105.00	105.00	105.00	105.00	105.00	105.00	105.00	105.00	105.00	110.00	105.00	110.00	105.00	113.00	105.00	113.00	105
4	Right	UCLA_2021_Pig5_RV_SS_2mA_40ms_20Hz_30onoff_postVagotomy_half_start2_002	2000	4000	67.00	67.00	67.00	67.00	67.00	67.00	67.00	67.00	68.00	67.00	68.00	67.00	68.80	67.00	68.80	67.00	69.50	67.00	69.50	67.00	67.00	67.00	67.00	67.00	67.00	67.00	67.00	67.00
5	Right	UCLA_2021_Pig6_RV_SS_2mA_40ms_20Hz_30onoff_vagotomy	2000	4000	103.50	103.50	103.50	103.50	103.50	103.50	103.50	103.50	103.50	103.50	103.20	103.70	103.70	103.70	103.70	103.70	103.00	103.50	103.00	103.50	103.20	103.50	103.50	103.50	103.50	103.50	103.50	103.50
6	Right	UCLA_2022_Pig2_42_Full_Vagus_7mA_1ms_POSTVAGOTOMY_OddPairs_Manual	7000	1000	58.00	56.00	58.00	56.00	64.00	56.00	64.00	56.00	67.00	56.00	67.00	56.00	68.00	56.00	68.00	56.00	65.00	56.00	65.00	56.00	65.00	56.00	65.00	56.00	65.00	56.00	65.00	56.00
7	Right	UCLA_2022_Pig3_45_sVNS_RV_Cardiac_5mA_1mS_OddPairs_POST_VAGOTOMY_BOTH_VAGI	5000	1000	97.00	97.00	97.00	97.00	101.00	97.00	101.00	97.00	100.00	97.00	100.00	97.00	97.00	97.00	97.00	97.00	101.00	97.00	101.00	97.00	105.00	97.00	105.00	97.00	97.00	97.00	97.00	97.00
8	Right	37_SS_RV_OddPairs_5mA_1ms	5000	1000	99.00	95.00	99.00	95.00	105.00	97.00	105.00	97.00	111.00	100.00	111.00	100.00	109.00	99.00	109.00	99.00	110.00	100.00	110.00	100.00	117.00	100.00	117.00	100.00	109.00	100.00	109.00	100.00
9	Right	UCLA_2022_Pig5_44_SS_RV_OddPairs_5mA_1ms	5000	1000	65.00	59.00	65.00	59.00	64.00	59.00	64.00	59.00	62.00	59.00	62.00	59.00	59.00	59.00	59.00	59.00	62.00	59.00	62.00	59.00	63.00	59.00	63.00	59.00	65.00	59.00	65.00	59.00
10	Right	UCLA_2022_Pig6_47_SS_RV_OddPairs_5mA_1ms_postVagotomy	5000	1000	77.00	72.00	77.00	72.00	73.00	72.00	73.00	72.00	74.00	72.00	74.00	72.00	74.00	72.00	74.00	72.00	74.00	72.00	74.00	72.00	74.00	72.00	74.00	72.00	74.00	72.00	74.00	72.00

Supplementary Table 2.3: Pulmonary sVNS Data

Animal Number	Vagus	Filename in Dataset	Pulse Features		Breathing Rate [Breath/min] - Stimulation Pair vs Off-Time																											
			Amplitude [µA]	Width [µs]	1	Off	2	Off	3	Off	4	Off	5	Off	6	Off	7	Off	8	Off	9	Off	10	Off	11	Off	12	Off	13	Off	14	Off
7	Right	08_sVNS_RV_Pulm_2000uA_200us	2000	200	26.9	32.3	21.8	32.5	21.6	31.4	21.7	33.1	18.8	34.6	22.9	34.0	22.0	33.1	16.9	33.6	19.9	34.8	25.6	34.5	23.8	33.2	24.8	35.0	34.0	34.8	33.2	33.8
8	Right	05_SS_RV_Pulm_15s_1p2mA	1200	50	27.1	32.0	27.4	33.1	19.3	32.6	28.4	31.9	5.1	30.1	17.9	30.3	18.8	30.2	28.6	30.8	33.4	33.1	35.2	34.1	35.6	34.3	35.8	34.6	35.5	34.0	34.2	34.8
9	Right	09_SS_RV_Pulm_1p5_repeat	1500	50	33.6	33.4	29.2	33.7	30.9	34.3	32.5	34.0	31.6	34.1	34.2	34.8	34.0	35.2	32.1	35.9	31.4	35.4	31.8	34.8	31.5	34.0	31.1	33.3	2.0	26.1	2.0	24.7
10	Right	09_SS_RV_Pulm_700uA_repeat_after_more_sedation	700	50	15.9	19.3	17.2	19.6	18.0	19.9	17.9	19.3	16.3	18.6	14.7	18.0	19.9	18.8	14.9	17.5	2.0	16.9	2.0	16.9	17.3	17.7	13.8	16.9	13.1	17.4	18.0	18.4

Section 3
Electrophysiology/selective nerve stimulation (sVNS) per nerve measurements:

In each of the following 10 tables (Tables 3.1 - 3.10), the relative variation between stimulation on and stimulation off (Val(stim)-Val(rest))/Val(rest)) for each of the respective readings (laryngeal EMG, pulmonary respiratory rate, cardiac heart rate) is shown for the recurrent laryngeal function (L), pulmonary function (P), cardiac efferent function (pre-vagotomy, C) and cardiac afferent function (post-vagotomy, CP).

The electrodes with their corresponding organotopic response were reorganized in each table with the most responsive electrode with respect to the cardiac pre-vagotomy/efferent (red) response positioned first in the rows.

L = Laryngeal (green), P = Pulmonary (blue), C = Cardiac, efferent/pre-vagotomy (red), CP = Cardiopulmonary, afferent/post-vagotomy (pink). Green blocks show the strongest, significant response, and yellow blocks show other significant responses per organ.

Supplementary Table 3.1

Nerve 6

Electrodes	Fascicle Type 'L'	'P'	'C'	CP'
	Relative variation of EMG (uV) between stim on and stim off [unitless]	Relative variation of RR (breaths per minute) between stim on and stim off [unitless]	Relative variation of HR (beats per minute) between stim on and stim off [unitless]	Relative variation of HR (bpm) between stim on and stim off [unitless]
**E3**	5.28		-0.12	0.14
**E4**	0		0.01	0.14
**E5**	-0.01		0	0.2
**E6**	-0.01		0.01	0.2
**E7**	0		0.01	0.21
**E8**	0.03		0	0.21
**E9**	0.02		0	0.16
**E10**	0		0.01	0.16
**E11**	675.9		0.01	0.16
**E12**	649.32		0	0.16
**E13**	79.7		-0.09	0.16
**E14**	56.05		-0.09	0.16
**E1**	5.97		-0.08	0.04
**E2**	6.16		-0.09	0.04
**max**	675.9	0	0.12	0.21

Supplementary Table 3.2

Nerve 7

Electrodes	Fascicle Type 'L'	'P'	'C'	CP'
	Relative variation of EMG (uV) between stim on and stim off [unitless]	Relative variation of RR (breaths per minute) between stim on and stim off [unitless]	Relative variation of HR (beats per minute) between stim on and stim off [unitless]	Relative variation of HR (bpm) between stim on and stim off [unitless]
**E11**	198.05	-0.3	-0.09	0.08
**E12**	408.78	-0.3	-0.09	0.08
**E13**	116.81	0	0.07	0
**E14**	3.26	0	0	0
**E1**	169.53	-0.2	0	0
**E2**	428.36	-0.3	-0.01	0
**E3**	376.94	-0.3	0	0.04
**E4**	98.24	-0.3	-0.01	0.04
**E5**	1.6	-0.5	-0.01	0.03
**E6**	-0.2	-0.3	-0.01	0.03
**E7**	-0.1	-0.3	-0.01	0
**E8**	-0.1	-0.5	-0.01	0
**E9**	3.65	-0.4	-0.01	0.04
**E10**	22.37	-0.3	-0.08	0.04
**max**	428.36	0.5	0.09	0.08

Supplementary Table 3.3

Nerve 8

Electrodes	Fascicle Type 'L'	'P'	'C'	CP'
	Relative variation of EMG (uV) between stim on and stim off [unitless]	Relative variation of RR (breaths per minute) between stim on and stim off [unitless]	Relative variation of HR (beats per minute) between stim on and stim off [unitless]	Relative variation of HR (beats per minute) between stim on and stim off [unitless]
**E11**		0	-0.09	0.18
**E12**		0	-0.04	0.18
**E13**		0	-0.01	0.09
**E14**		0	0	0.09
**E1**		-0.2	-0.04	0.05
**E2**		-0.2	-0.03	0.05
**E3**		-0.4	-0.01	0.08
**E4**		-0.1	-0.01	0.08
**E5**		-0.8	-0.01	0.1
**E6**		-0.4	-0.01	0.1
**E7**		-0.4	-0.01	0.09
**E8**		-0.1	-0.01	0.09
**E9**		0	-0.01	0.09
**E10**		0	-0.08	0.09
**max**	0	0.83	0.09	0.18

Supplementary Table 3.4

Nerve 9

Electrodes	Fascicle Type 'L'	'P'	'C'	CP'
	Relative variation of EMG (uV) between stim on and stim off [unitless]	Relative variation of RR (breaths per minute) between stim on and stim off [unitless]	Relative variation of HR (beats per minute) between stim on and stim off [unitless]	Relative variation of HR (beats per minute) between stim on and stim off [unitless]
**E12**	3.25	-0.1	-0.1	0.07
**E13**	0.09	-0.9	-0.03	0.1
**E14**	0.04	-0.9	-0.02	0.1
**E1**	0.09	0	-0.06	0.1
**E2**	0.22	-0.1	-0.03	0.1
**E3**	0.8	-0.1	-0.01	0.08
**E4**	4.38	0	-0.03	0.08
**E5**	17.29	-0.1	-0.05	0.05
**E6**	0.02	0	0	0.05
**E7**	0.01	0	-0.01	0
**E8**	19.38	-0.1	-0.08	0
**E9**	10.84	-0.1	-0.08	0.05
**E10**	17.81	-0.1	-0.08	0.05
**E11**	13.59	-0.1	-0.08	0.07
**max**	19.38	0.92	0.1	0.1

Supplementary Table 3.5

Nerve 10

Electrodes	Fascicle Type: L	P	C	CP
	Relative variation of EMG (uV) between stim on and stim off [unitless]	Relative variation of RR (breaths per minute) between stim on and stim off [unitless]	Relative variation of HR (beats per minute) between stim on and stim off [unitless]	Relative variation of HR (beats per minute) between stim on and stim off [unitless]
**E10**	85.55	-0.9	-0.06	0.03
**E11**	121.07	0	-0.05	0.03
**E12**	206.66	-0.2	-0.04	0.03
**E13**	66.32	-0.2	-0.02	0.03
**E14**	0.15	0	-0.01	0.03
**E1**	27.32	-0.2	0	0.07
**E2**	24.1	-0.1	0	0.07
**E3**	55.66	-0.1	0	0.01
**E4**	159.53	-0.1	-0.01	0.01
**E5**	71.39	-0.1	0	0.03
**E6**	0.13	-0.2	-0.01	0.03
**E7**	0.81	0.1	0	0.03
**E8**	0.99	-0.1	-0.03	0.03
**E9**	18.18	-0.9	0	0.03
**max**	206.66	0.88	0.06	0.07

Supplementary Table 3.6

Nerve 1

Electrodes	Fascicle Type: 'L'	'P'	'C'	CP'
	Relative variation of EMG (uV) between stim on and stim [unitless]	Relative variation of RR (breaths per minute) between stim on and stim off [unitless]	Relative variation of HR (beats per minute) between stim on and stim off [unitless]	Relative variation of HR (beats per minute) between stim on and stim off [unitless]
**E1**	1.1		-0.12	0
**E2**	5.49		-0.11	0
**E3**	4.48		-0.09	0
**E4**	3.4		-0.07	0
**E5**	2.93		0	0
**E6**	1.49		0	-0.01
**E7**	0.36		0	-0.01
**E8**	-0.25		0	-0.02
**E9**	0.26		0	-0.01
**E10**	0.25		0	0
**E11**	0.31		0	-0.01
**E12**	1.15		-0.09	0
**E13**	0.8		-0.1	0
**E14**	0.12		0	0
**max**	5.49	0	0.12	0.02

Supplementary Table 3.7

Nerve 2

Electrodes	Fascicle Type: 'L'	'P'	'C'	CP'
	Relative variation of EMG (uV) between stim on and stim off [unitless]	Relative variation of RR (breaths per minute) between stim on and stim off [unitless]	Relative variation of HR (beats per minute) between stim on and stim off [unitless]	Relative variation of HR (beats per minute) between stim on and stim off [unitless]
E2	1.94		-0.16	0
E3	0.76		-0.08	-0.01
E4	0.13		-0.01	-0.03
E5	0.13		0	-0.03
E6	0.01		0	-0.02
E7	0		0	-0.01
E8	0.11		0	0
E9	0.21		0	0
E10	0.43		-0.01	0
E11	-0.02		0	0
E12	0.05		0	0
E13	0.01		0	0
E14	0.08		0	0
E1	1.5		-0.09	0
max	1.94	0	0.16	0.03

Supplementary Table 3.8

Nerve 3

Electrodes	Fascicle Type: 'L'	'P'	'C'	CP'
	Relative variation of EMG (uV) between stim on and stim off [unitless]	Relative variation of RR (breaths per minute) between stim on and stim off [unitless]	Relative variation of HR (beats per minute) between stim on and stim off [unitless]	Relative variation of HR (beats per minute) between stim on and stim off [unitless]
E6	24.66		-0.03	0
E7	21.58		-0.02	0
E8	6.6		-0.02	0
E9	2.62		0	0
E10	0.33		0	0
E11	0.23		0	0.05
E12	0.1		0	0.05
E13	0.02		0	0.08
E14	0.04		0	0.08
E1	0.07		0	0.08
E2	1.12		-0.01	0.08
E3	3.59		0	0
E4	13.03		-0.01	0
E5	22.89		-0.02	0
max	24.66	0	0.03	0.08

Supplementary Table 3.9

Nerve 4

Electrodes	Fascicle Type: 'L'	'P'	'C'	CP'
	Relative variation of EMG (uV) between stim on and stim off [unitless]	Relative variation of RR (breaths per minute) between stim on and stim off [unitless]	Relative variation of HR (beats per minute) between stim on and stim off [unitless]	Relative variation of HR (beats per minute) between stim on and stim off [unitless]
**E13**	6.67		-0.09	0
**E14**	4.31		-0.01	0
**E1**	4.27		-0.05	0
**E2**	6.8		-0.06	0
**E3**	9.18		0	0
**E4**	1.18		0	0
**E5**	0.22		-0.01	0.01
**E6**	-0.14		0	0.01
**E7**	-0.22		0	0.03
**E8**	0.02		-0.01	0.03
**E9**	-0.27		-0.01	0.04
**E10**	-0.46		-0.01	0.04
**E11**	0.69		0	0
**E12**	1.81		-0.07	0
**max**	9.18	0	0.09	0.04

Supplementary Table 3.10

Nerve 5

Electrodes	Fascicle Type: 'L'	'P'	'C'	CP'
	Relative variation of EMG (uV) between stim on and stim off [unitless]	Relative variation of RR (breaths per minute) between stim on and stim off [unitless]	Relative variation of HR (beats per minute) between stim on and stim off [unitless]	Relative variation of HR (beats per minute) between stim on and stim off [unitless]
**E3**	13.01		-0.04	0
**E4**	8.71		-0.03	0
**E5**	0.06		-0.02	0
**E6**	-0.05		0	0
**E7**	-0.02		0	0
**E8**	-0.05		0	0
**E9**	-0.05		0	0
**E10**	0		0	0
**E11**	-0.09		0	0
**E12**	0.64		0	0
**E13**	8.01		0	0
**E14**	5.39		0	0
**E1**	5.15		0	0
**E2**	8.91		-0.01	0
**max**	13.01	0	0.04	0

In each of the following 10 tables (Tables 3.11 - 3.20), the ***normalized*** *relative variation between stimulation on and stimulation off for each of the respective readings (laryngeal EMG, pulmonary respiratory rate, cardiac heart rate) is shown for the recurrent laryngeal function (L), pulmonary function (P), cardiac efferent function (pre-vagotomy, C) and cardiac afferent function (post-vagotomy, CP) with the highest response as 1 and rest of the values normalized to 1.*

L = Laryngeal (green), P = Pulmonary (blue), C = Cardiac, efferent/pre-vagotomy (red), CP = Cardiopulmonary, afferent/post-vagotomy (pink).

Supplementary Table 3.11

Nerve 6

Angle of Electrode	Electrodes	Fascicle Type: 'L'	'P'	'C'	CP'
		Normalized relative variation of EMG (uV) between stim on and stim off [unitless]	Normalized relative variation of RR (breaths per minute) between stim on and stim off [unitless]	Normalized relative variation of HR (beats per minute) between stim on and stim off [unitless]	Normalized relative variation of HR (beats per minute) between stim on and stim off [unitless]
25.71429	1	0.01		1	0.67
51.42857	14	0		0.12	0.67
77.14286	13	0		0.03	0.92
102.8571	12	0		0.06	0.92
128.5714	11	0		0.05	1
154.2857	10	0		0.01	1
180	9	0		0.03	0.75
205.7143	8	0		0.06	0.75
231.4286	7	1		0.09	0.75
257.1429	6	0.96		0	0.75
282.8571	5	0.12		0.72	0.75
308.5714	4	0.08		0.68	0.75
334.2857	3	0.01		0.6	0.17
360	2	0.01		0.71	0.17

Supplementary Table 3.12

Nerve 7

Angle of Electrode	Electrodes	Fascicle Type: L	P	C	CP
		Normalized relative variation of EMG (uV) between stim on and stim off [unitless]	Normalized relative variation of RR (breaths per minute) between stim on and stim off [unitless]	Normalized relative variation of HR (beats per minute) between stim on and stim off [unitless]	Normalized relative variation of HR (beats per minute) between stim on and stim off [unitless]
25.71429	1	0.46	0.57	0.96	1
51.42857	14	0.95	0.59	1	1
77.14286	13	0.27	0.05	0.78	0
102.8571	12	0.01	0.03	0.01	0
128.5714	11	0.4	0.34	0.03	0
154.2857	10	1	0.66	0.08	0
180	9	0.88	0.63	0	0.5
205.7143	8	0.23	0.69	0.08	0.5
231.4286	7	0	0.92	0.07	0.38
257.1429	6	0	0.66	0.1	0.38
282.8571	5	0	0.68	0.11	0
308.5714	4	0	1	0.1	0
334.2857	3	0.01	0.86	0.13	0.5
360	2	0.05	0.52	0.89	0.5

Supplementary Table 3.13

Nerve 8

Angle of Electrode	Electrodes	**Fascicle Type** 'L'	'P'	'C'	CP'
		Normalized relative variation of EMG (uV) between stim on and stim off [unitless]	Normalized relative variation of RR (breaths per minute) between stim on and stim off [unitless]	Normalized relative variation of HR (beats per minute) between stim on and stim off [unitless]	Normalized relative variation of HR (beats per minute) between stim on and stim off [unitless]
25.71429	1		0.05	1	1
51.42857	14		0.04	0.43	1
77.14286	13		0.05	0.07	0.53
102.8571	12		0.02	0.02	0.53
128.5714	11		0.18	0.4	0.28
154.2857	10		0.21	0.29	0.28
180	9		0.49	0.15	0.47
205.7143	8		0.13	0.07	0.47
231.4286	7		1	0.07	0.56
257.1429	6		0.49	0.05	0.56
282.8571	5		0.45	0.09	0.53
308.5714	4		0.08	0.06	0.53
334.2857	3		0.01	0.13	0.53
360	2		0.04	0.82	0.53

Supplementary Table 3.14

Nerve 9

Angle of Electrode	Electrodes	**Fascicle Type** 'L'	'P'	'C'	CP'
		Normalized relative variation of EMG (uV) between stim on and stim off [unitless]	Normalized relative variation of RR (breaths per minute) between stim on and stim off [unitless]	Normalized relative variation of HR (beats per minute) between stim on and stim off [unitless]	Normalized relative variation of HR (beats per minute) between stim on and stim off [unitless]
25.71429	1	0.17	0.07	1	0.67
51.42857	14	0	1	0.27	1
77.14286	13	0	1	0.19	1
102.8571	12	0	0.01	0.55	1
128.5714	11	0.01	0.14	0.29	1
154.2857	10	0.04	0.11	0.14	0.83
180	9	0.23	0.05	0.27	0.83
205.7143	8	0.89	0.08	0.5	0.5
231.4286	7	0	0.02	0.01	0.5
257.1429	6	0	0.04	0.1	0
282.8571	5	1	0.12	0.83	0
308.5714	4	0.56	0.12	0.81	0.5
334.2857	3	0.92	0.09	0.81	0.5
360	2	0.7	0.08	0.79	0.67

Supplementary Table 3.15

Nerve 10

		Fascicle Type			
**Angle of Electrode**	**Electrodes**	**L**	**P**	**C**	**CP**
		Normalized relative variation of EMG (uV) between stim on and stim off [unitless]	Normalized relative variation of RR (breaths per minute) between stim on and stim off [unitless]	Normalized relative variation of HR (beats per minute) between stim on and stim off [unitless]	Normalized relative variation of HR (beats per minute) between stim on and stim off [unitless]
25.71429	1	0.41	1	1	0.4
51.42857	14	0.59	0.03	0.83	0.4
77.14286	13	1	0.21	0.67	0.4
102.8571	12	0.32	0.28	0.33	0.4
128.5714	11	0	0.03	0.09	0.4
154.2857	10	0.13	0.2	0.02	1
180	9	0.12	0.14	0	1
205.7143	8	0.27	0.11	0.03	0.2
231.4286	7	0.77	0.09	0.1	0.2
257.1429	6	0.35	0.14	0.04	0.4
282.8571	5	0	0.21	0.14	0.4
308.5714	4	0	0.07	0	0.4
334.2857	3	0	0.16	0.5	0.4
360	2	0.09	1	0.02	0.4

Supplementary Table 3.16

Nerve 1

		Fascicle Type			
**Angle of Electrode**	**Electrodes**	**L**	**P**	**C**	**CP**
		Normalized relative variation of EMG (uV) between stim on and stim off [unitless]	Normalized relative variation of RR (breaths per minute) between stim on and stim off [unitless]	Normalized relative variation of HR (beats per minute) between stim on and stim off [unitless]	Normalized relative variation of HR (beats per minute) between stim on and stim off [unitless]
25.71429	1	0.2		1	0
51.42857	14	1		0.92	0
77.14286	13	0.82		0.76	0
102.8571	12	0.62		0.56	0
128.5714	11	0.53		0.03	0.25
154.2857	10	0.27		0	0.45
180	9	0.07		0	0.75
205.7143	8	0.05		0.01	1
231.4286	7	0.05		0	0.75
257.1429	6	0.05		0	0.25
282.8571	5	0.06		0.01	0.5
308.5714	4	0.21		0.76	0
334.2857	3	0.15		0.84	0
360	2	0.02		0.01	0

Supplementary Table 3.17

Nerve 2

		Fascicle Type			
**Angle of Electrode**	**Electrodes**	**L**	**P**	**C**	**CP**
		Normalized relative variation of EMG (uV) between stim on and stim off [unitless]	Normalized relative variation of RR (breaths per minute) between stim on and stim off [unitless]	Normalized relative variation of HR (beats per minute) between stim on and stim off [unitless]	Normalized relative variation of HR (beats per minute) between stim on and stim off [unitless]
25.71429	1	1		1	0
51.42857	14	0.39		0.53	0.25
77.14286	13	0.07		0.08	1
102.8571	12	0.07		0.02	1
128.5714	11	0.01		0.01	0.75
154.2857	10	0		0.01	0.5
180	9	0.06		0	0
205.7143	8	0.11		0.01	0
231.4286	7	0.22		0.08	0
257.1429	6	0.01		0.01	0
282.8571	5	0.03		0.02	0
308.5714	4	0		0.02	0
334.2857	3	0.04		0.02	0
360	2	0.78		0.58	0

Supplementary Table 3.18

Nerve 3

		Fascicle Type			
**Angle of Electrode**	**Electrodes**	**L**	**P**	**C**	**CP**
		Normalized relative variation of EMG (uV) between stim on and stim off [unitless]	Normalized relative variation of RR (breaths per minute) between stim on and stim off [unitless]	Normalized relative variation of HR (beats per minute) between stim on and stim off [unitless]	Normalized relative variation of HR (beats per minute) between stim on and stim off [unitless]
25.71429	1	1		1	0
51.42857	14	0.88		0.93	0
77.14286	13	0.27		0.86	0
102.8571	12	0.11		0.08	0
128.5714	11	0.01		0.02	0
154.2857	10	0.01		0.01	0.63
180	9	0		0.01	0.63
205.7143	8	0		0.09	1
231.4286	7	0		0.03	1
257.1429	6	0		0.06	1
282.8571	5	0.05		0.3	1
308.5714	4	0.15		0.18	0
334.2857	3	0.53		0.45	0
360	2	0.93		0.76	0

Supplementary Table 3.19

Nerve 4

Angle of Electrode	Electrodes	Fascicle Type: L	P	C	CP
		Normalized relative variation of EMG (uV) between stim on and stim off [unitless]	Normalized relative variation of RR (breaths per minute) between stim on and stim off [unitless]	Normalized relative variation of HR (beats per minute) between stim on and stim off [unitless]	Normalized relative variation of HR (beats per minute) between stim on and stim off [unitless]
25.71429	1	0.73		1	0
51.42857	14	0.47		0.13	0
77.14286	13	0.47		0.63	0
102.8571	12	0.74		0.75	0
128.5714	11	1		0.05	0
154.2857	10	0.13		0.05	0
180	9	0.02		0.15	0.4
205.7143	8	0.02		0.03	0.4
231.4286	7	0.02		0.02	0.72
257.1429	6	0		0.07	0.72
282.8571	5	0.03		0.06	1
308.5714	4	0.05		0.07	1
334.2857	3	0.07		0	0
360	2	0.2		0.79	0

Supplementary Table 3.20

Nerve 5

Angle of Electrode	Electrodes	Fascicle Type: L	P	C	CP
		Normalized relative variation of EMG (uV) between stim on and stim off [unitless]	Normalized relative variation of RR (breaths per minute) between stim on and stim off [unitless]	Normalized relative variation of HR (beats per minute) between stim on and stim off [unitless]	Normalized relative variation of HR (beats per minute) between stim on and stim off [unitless]
25.71429	1	1		1	0
51.42857	14	0.67		0.78	0
77.14286	13	0		0.43	0
102.8571	12	0		0.03	1
128.5714	11	0		0.01	0
154.2857	10	0		0.01	0
180	9	0		0.01	1
205.7143	8	0		0.02	1
231.4286	7	0.01		0.01	0.6
257.1429	6	0.05		0	0
282.8571	5	0.62		0.02	0
308.5714	4	0.41		0.01	0
334.2857	3	0.4		0.02	0
360	2	0.68		0.13	0

Section 4
MicroCT per nerve measurements:

The pigs and nerves are numbered 6-10 (corresponding with supplementary tables and figures 4.1.x – 4.5.x, respectively), which correlates with the nerve numbers in Table 1 within the text, and corresponding with the numbering used for the electrophysiology/sVNS data.

Per nerve, there are:
1. *the perimeter and area values for the mid-cervical cross section* (Supplementary Tables 4.1.1, 4.2.1, 4.3.1, 4.4.1, and 4.5.1)
2. *the traced and labeled cross section figure* (Supplementary Figures 4.1.1, 4.2.1, 4.3.1, 4.4.1, and 4.5.1)
3. *this figure plotted on a cartesian plane in order to determine the coordinates/position of each fascicle with respect to the nerve cross section and the electrode positions* (Supplementary Figures 4.1.2, 4.2.2, 4.3.2, 4.4.2, and 4.5.2)
4. *a graph showing the number and type of fascicles less than 0.5mm away from each electrode* (Supplementary Figures 4.1.3, 4.2.3, 4.3.3, 4.4.3, and 4.5.3)
5. *a table of the distance (mm) of each fascicle (and its type) within the cross section from each electrode around the circumference of the nerve* (Supplementary Tables 4.1.2, 4.2.2, 4.3.2, 4.4.2, and 4.5.2)
6. *a table showing the number of fascicles of each type within 0.5mm of each electrode around the nerve* (Supplementary Tables 4.1.3, 4.2.3, 4.3.3, 4.4.3, and 4.5.3)
7. *a table showing the previous table in %* (Supplementary Tables 4.1.4, 4.2.4, 4.3.4, 4.4.4, and 4.5.4)
8. *a table showing these values reordered with cardiac at the top (to correspond with the sVNS data)* (Supplementary Tables 4.1.5, 4.2.5, 4.3.5, 4.4.5, and 4.5.5)
9. *a table showing the previous table/order normalized to 1 (yellow blocks)* (Supplementary Tables 4.1.6, 4.2.6, 4.3.6, 4.4.6, and 4.5.6)

L = Laryngeal (green), P = Pulmonary (blue), C = Cardiac (red), CP = Cardiopulmonary (pink), LP = Laryngeal and pulmonary (turquoise), Aff = Afferent/sensory (orange), Eff = Efferent/motor (yellow), Mixed = afferent and efferent (purple).
In tables with color shading – shading is according to max (orange) and min (pale yellow).

Key for cross section figures:

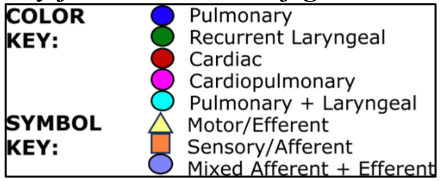

Nerve 6:

Supplementary Table 4.1.1

**Perimeter (mm)**	6.088699
**Area (mm2)**	2.03025

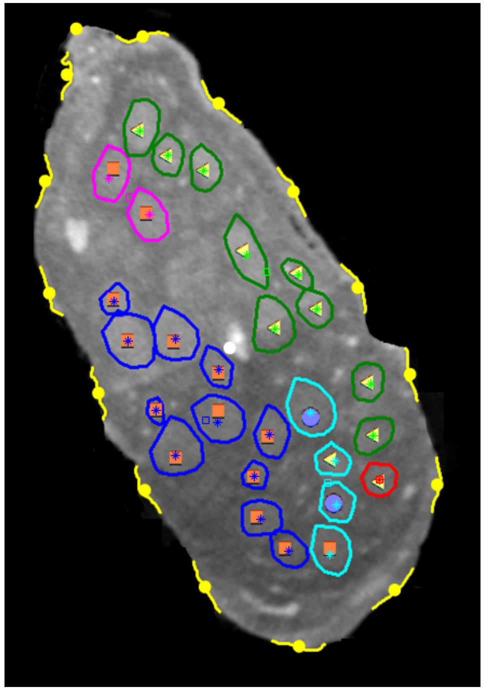

Supplementary Figure 4.1.1
Traced and labeled cross section of nerve 6

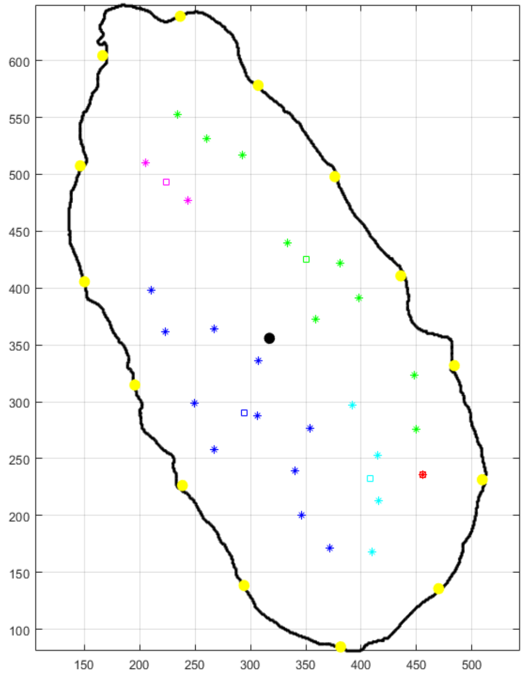

Supplementary Figure 4.1.2
Traced cross section mapped to determine co-ordinates and distance of fascicle centers of mass from the 14 electrodes for nerve 6

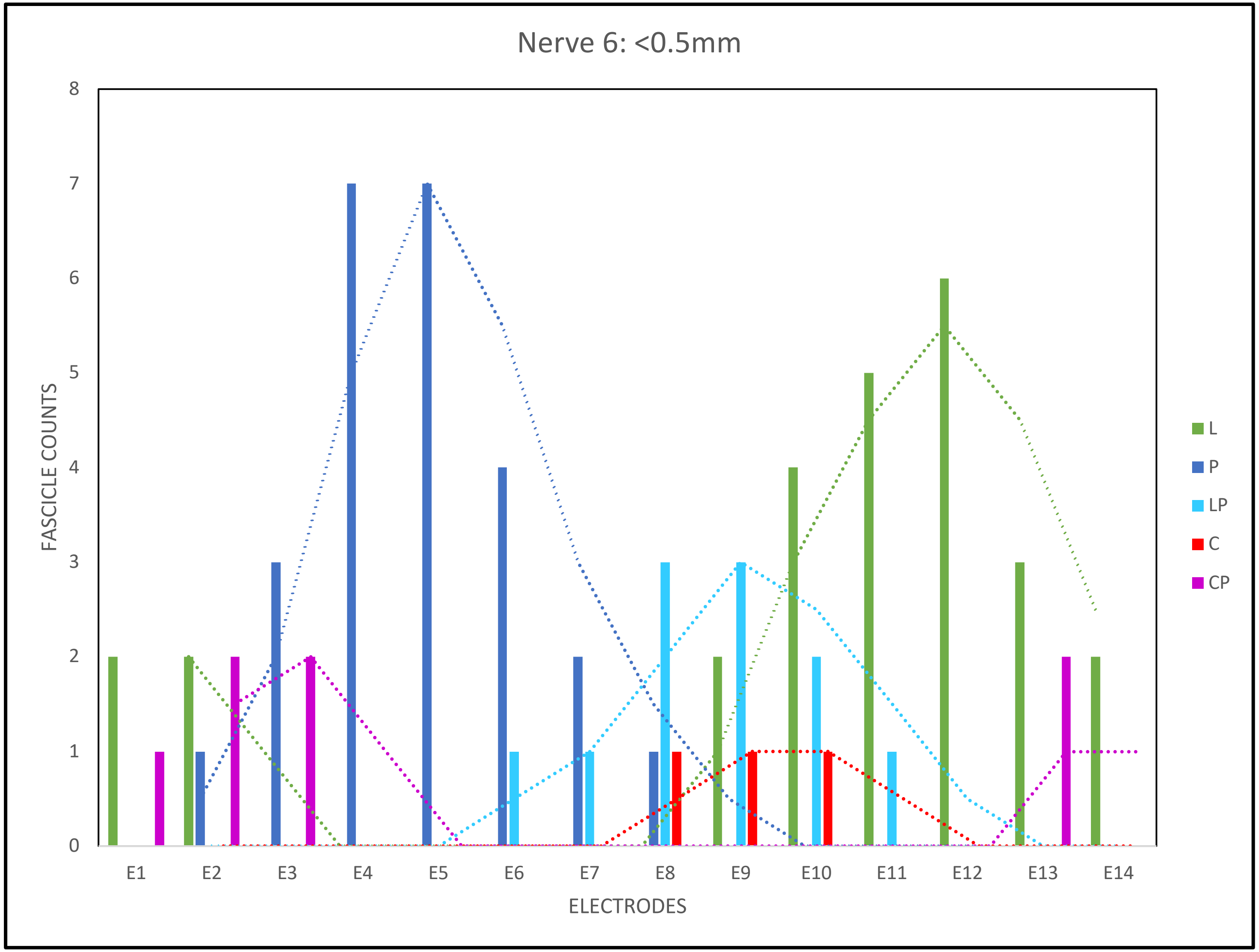

Supplementary Figure 4.1.3

A graph of the number and type of fascicles less than 0.5mm away from each electrode for nerve 6

Supplementary Table 4.1.2

The distance (mm) of each fascicle (and its type) within the cross section from each electrode around the circumference of the nerve (nerve 6)

Fascicle Progressive	1	2	3	4	5	6	7	8	9	10	11	12	13	14	15	16	17	18	19	20	21	22	23	24	25	26	27
Fascicle Type - Num	1	1	1	1	1	1	1	1	1	2	2	2	2	2	2	2	2	2	2	2	3	3	3	3	4	5	5
Fascicle Type - Char	'L'	'L'	'L'	'L'	'L'	'L'	'L'	'L'	'L'	'P'	'P'	'P'	'P'	'P'	'P'	'P'	'P'	'P'	'P'	'P'	'LP'	'LP'	'LP'	'LP'	'C'	CP'	CP'
E1	0.321	0.450	0.582	0.886	1.066	1.191	1.139	1.506	1.642	0.798	0.942	0.986	1.146	1.308	1.197	1.365	1.428	1.530	1.674	1.815	1.443	1.628	1.756	1.891	1.773	0.386	0.562
E2	0.374	0.440	0.557	0.752	0.945	1.050	0.952	1.338	1.445	0.480	0.622	0.710	0.889	1.027	0.880	1.049	1.174	1.253	1.387	1.533	1.224	1.401	1.511	1.627	1.559	0.223	0.384
E3	0.642	0.631	0.686	0.704	0.876	0.940	0.800	1.170	1.236	0.229	0.322	0.470	0.650	0.739	0.550	0.713	0.912	0.956	1.075	1.222	1.004	1.157	1.242	1.333	1.324	0.446	0.444
E4	0.913	0.854	0.849	0.704	0.811	0.819	0.657	0.956	0.974	0.320	0.207	0.328	0.430	0.430	0.211	0.346	0.617	0.617	0.716	0.861	0.747	0.863	0.919	0.983	1.030	0.739	0.640
E5	1.236	1.155	1.119	0.884	0.916	0.867	0.718	0.873	0.822	0.658	0.516	0.532	0.489	0.346	0.277	0.161	0.477	0.387	0.419	0.547	0.639	0.675	0.674	0.686	0.824	1.080	0.948
E6	1.585	1.491	1.432	1.150	1.122	1.033	0.920	0.909	0.786	1.032	0.887	0.859	0.749	0.567	0.631	0.464	0.571	0.418	0.304	0.319	0.705	0.630	0.540	0.452	0.715	1.445	1.295
E7	1.858	1.751	1.670	1.357	1.277	1.161	1.095	0.937	0.770	1.352	1.209	1.143	0.992	0.821	0.954	0.786	0.736	0.605	0.457	0.329	0.805	0.650	0.503	0.334	0.639	1.743	1.575
E8	1.814	1.693	1.590	1.263	1.134	1.004	0.991	0.713	0.536	1.397	1.267	1.156	0.977	0.847	1.040	0.897	0.692	0.628	0.528	0.394	0.678	0.490	0.357	0.258	0.383	1.735	1.551
E9	1.602	1.475	1.355	1.033	0.869	0.736	0.781	0.417	0.281	1.296	1.191	1.045	0.862	0.799	1.018	0.923	0.613	0.642	0.630	0.568	0.509	0.366	0.360	0.446	0.203	1.561	1.371
E10	1.263	1.135	1.007	0.703	0.518	0.396	0.499	0.141	0.248	1.067	0.995	0.831	0.671	0.694	0.898	0.868	0.534	0.649	0.723	0.742	0.373	0.397	0.518	0.680	0.378	1.253	1.065
E11	0.934	0.806	0.673	0.404	0.212	0.161	0.324	0.335	0.513	0.856	0.826	0.663	0.563	0.676	0.823	0.861	0.593	0.744	0.867	0.939	0.461	0.602	0.752	0.924	0.666	0.950	0.771
E12	0.577	0.457	0.323	0.273	0.288	0.413	0.477	0.716	0.885	0.734	0.775	0.654	0.666	0.838	0.893	0.997	0.840	0.989	1.133	1.237	0.763	0.938	1.089	1.255	1.036	0.649	0.510
E13	0.292	0.252	0.238	0.532	0.654	0.788	0.801	1.103	1.265	0.774	0.878	0.825	0.917	1.098	1.079	1.221	1.154	1.290	1.439	1.560	1.112	1.297	1.442	1.600	1.412	0.464	0.453
E14	0.325	0.418	0.509	0.837	0.986	1.120	1.108	1.439	1.593	0.917	1.049	1.047	1.177	1.354	1.287	1.446	1.440	1.563	1.712	1.843	1.421	1.609	1.749	1.899	1.736	0.502	0.613

Supplementary Table 4.1.3

The number of fascicles of each type within 0.5mm of each electrode around the nerve (nerve 6)

Pig	Threshold	Electrodes	Fascicle Type				
			L	P	LP	C	CP
6	0.5mm	E1	2.0	0.0	0.0	0.0	1.0
		E2	2.0	1.0	0.0	0.0	2.0
		E3	0.0	3.0	0.0	0.0	2.0
		E4	0.0	7.0	0.0	0.0	0.0
		E5	0.0	7.0	0.0	0.0	0.0
		E6	0.0	4.0	1.0	0.0	0.0
		E7	0.0	2.0	1.0	0.0	0.0
		E8	0.0	1.0	3.0	1.0	0.0
		E9	2.0	0.0	3.0	1.0	0.0
		E10	4.0	0.0	2.0	1.0	0.0
		E11	5.0	0.0	1.0	0.0	0.0
		E12	6.0	0.0	0.0	0.0	0.0
		E13	3.0	0.0	0.0	0.0	2.0
		E14	2.0	0.0	0.0	0.0	0.0

Supplementary Table 4.1.4

The number of fascicles of each type within 0.5mm of each electrode around the nerve in % (nerve 6)

Pig	Threshold	Electrodes	Fascicle Type (%)				
			L	P	LP	C	CP
6	0.5mm	E1	22.2	0.0	0.0	0.0	50.0
		E2	22.2	9.1	0.0	0.0	100.0
		E3	0.0	27.3	0.0	0.0	100.0
		E4	0.0	63.6	0.0	0.0	0.0
		E5	0.0	63.6	0.0	0.0	0.0
		E6	0.0	36.4	25.0	0.0	0.0
		E7	0.0	18.2	25.0	0.0	0.0
		E8	0.0	9.1	75.0	100.0	0.0
		E9	22.2	0.0	75.0	100.0	0.0
		E10	44.4	0.0	50.0	100.0	0.0
		E11	55.6	0.0	25.0	0.0	0.0
		E12	66.7	0.0	0.0	0.0	0.0
		E13	33.3	0.0	0.0	0.0	100.0
		E14	22.2	0.0	0.0	0.0	0.0

Supplementary Table 4.1.5

The number of fascicles of each type within 0.5mm of each electrode around the nerve reordered with cardiac at the top (to correspond with sVNS data) (nerve 6)

	Fascicle Type			
**Electrodes**	**'L'**	**'P'**	**'C'**	**CP'**
**E8**	0	1	1	0
**E9**	2	0	1	0
**E10**	4	0	1	0
**E11**	5	0	0	0
**E12**	6	0	0	0
**E13**	3	0	0	2
**E14**	2	0	0	0
**E1**	2	0	0	1
**E2**	2	1	0	2
**E3**	0	3	0	2
**E4**	0	7	0	0
**E5**	0	7	0	0
**E6**	0	4	0	0
**E7**	0	2	0	0
**max**	6	7	1	2

Supplementary Table 4.1.6

Supplementary Table 4.1.5 values normalized to 1 (yellow blocks) (nerve 6)

		Fascicle Type			
**Angle of Electrode**	**Electrodes**	**'L'**	**'P'**	**'C'**	**CP'**
25.71428571	1	0	0.142857	1	0
51.42857143	2	0.333333	0	1	0
77.14285714	3	0.666667	0	1	0
102.8571429	4	0.833333	0	0	0
128.5714286	5	1	0	0	0
154.2857143	6	0.5	0	0	1
180	7	0.333333	0	0	0
205.7142857	8	0.333333	0	0	0.5
231.4285714	9	0.333333	0.142857	0	1
257.1428571	10	0	0.428571	0	1
282.8571429	11	0	1	0	0
308.5714286	12	0	1	0	0
334.2857143	13	0	0.571429	0	0
360	14	0	0.285714	0	0

Nerve 7:

Supplementary Table 4.2.1

**Perimeter (mm)**	6.845509
**Area (mm2)**	3.07051

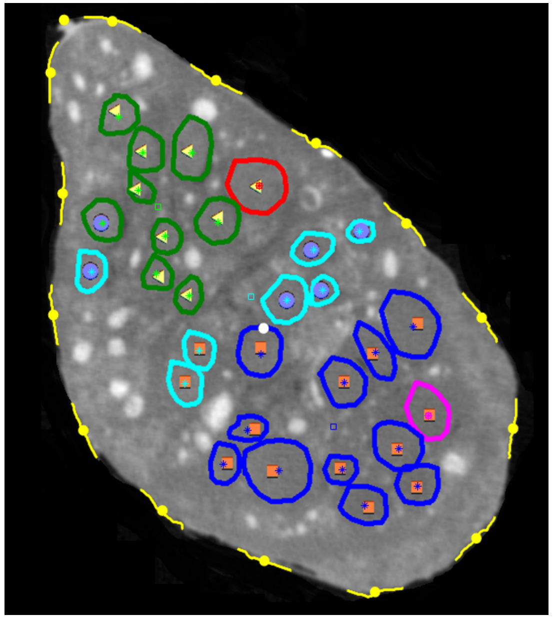

Supplementary Figure 4.2.1
Traced and labeled cross section of nerve 7

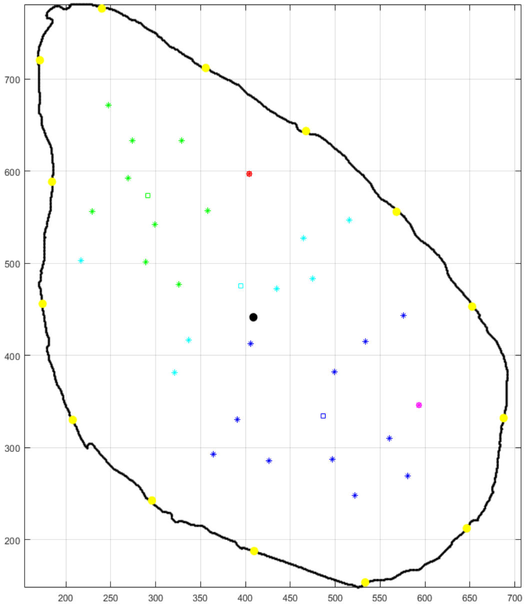

Supplementary Figure 4.2.2
Traced cross section mapped to determine co-ordinates and distance of fascicle centers of mass from the 14 electrodes for nerve 7

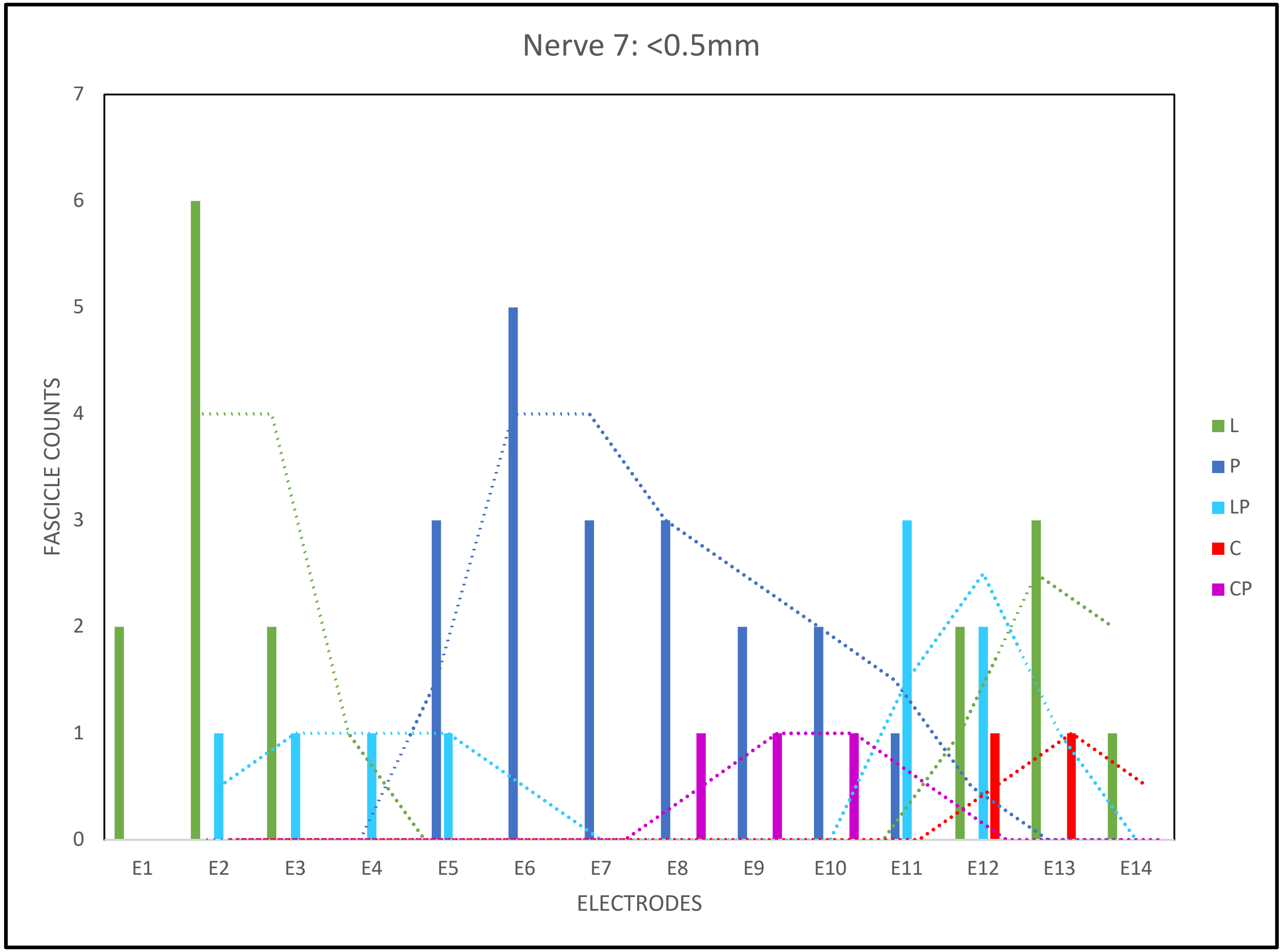

Supplementary Figure 4.2.3

A graph of the number and type of fascicles less than 0.5mm away from each electrode for nerve 7

Supplementary Table 4.2.2

The distance (mm) of each fascicle (and its type) within the cross section from each electrode around the circumference of the nerve (nerve 7)

Fascicle Progressive	1	2	3	4	5	6	7	8	9	10	11	12	13	14	15	16	17	18	19	20	21	22	23	24	25	26	27	28	29
Fascicle Type - Num	1	1	1	1	1	1	1	1	1	2	2	2	2	2	2	2	2	2	2	2	3	3	3	3	3	3	3	4	5
Fascicle Type - Char	'L'	'L'	'L'	'L'	'L'	'L'	'L'	'L'	'L'	'P'	'P'	'P'	'P'	'P'	'P'	'P'	'P'	'P'	'P'	'P'	'LP'	'LP'	'LP'	'LP'	'LP'	'LP'	'LP'	'C'	CP'
E1	0.311	0.467	0.624	0.558	0.603	0.759	0.858	0.861	0.997	1.337	1.629	1.639	1.696	1.549	1.621	1.741	1.874	2.034	1.954	2.107	0.768	1.282	1.195	1.253	1.333	1.216	1.334	0.911	1.950
E2	0.360	0.345	0.523	0.292	0.191	0.427	0.609	0.471	0.623	0.977	1.300	1.348	1.443	1.143	1.195	1.338	1.501	1.657	1.616	1.760	0.316	0.858	0.793	0.955	1.068	0.992	1.155	0.759	1.642
E3	0.788	0.703	0.813	0.573	0.394	0.524	0.725	0.427	0.530	0.815	1.152	1.252	1.390	0.867	0.865	1.050	1.260	1.401	1.426	1.548	0.220	0.570	0.579	0.904	1.044	1.035	1.223	0.932	1.497
E4	1.190	1.072	1.128	0.930	0.785	0.798	0.941	0.655	0.652	0.743	1.023	1.166	1.332	0.634	0.556	0.770	1.011	1.123	1.220	1.308	0.599	0.430	0.539	0.927	1.065	1.120	1.303	1.146	1.333
E5	1.494	1.352	1.354	1.211	1.108	1.035	1.108	0.894	0.817	0.701	0.852	1.016	1.191	0.447	0.293	0.474	0.712	0.782	0.942	0.990	0.941	0.486	0.620	0.927	1.036	1.144	1.298	1.281	1.087
E6	1.766	1.609	1.564	1.479	1.418	1.283	1.289	1.160	1.041	0.779	0.739	0.895	1.053	0.496	0.397	0.345	0.457	0.441	0.670	0.655	1.277	0.735	0.831	0.987	1.045	1.188	1.295	1.415	0.837
E7	2.047	1.884	1.801	1.769	1.744	1.568	1.520	1.468	1.328	0.999	0.798	0.903	1.011	0.783	0.758	0.589	0.478	0.329	0.548	0.431	1.629	1.075	1.135	1.152	1.156	1.312	1.361	1.596	0.696
E8	2.105	1.942	1.822	1.850	1.869	1.656	1.554	1.588	1.437	1.083	0.777	0.801	0.834	0.972	1.015	0.804	0.578	0.448	0.451	0.300	1.792	1.267	1.282	1.158	1.108	1.256	1.242	1.572	0.498
E9	1.923	1.768	1.618	1.703	1.764	1.527	1.379	1.496	1.346	1.013	0.674	0.603	0.544	1.025	1.127	0.918	0.677	0.642	0.448	0.428	1.730	1.278	1.247	0.998	0.901	1.023	0.951	1.341	0.331
E10	1.593	1.449	1.280	1.410	1.506	1.260	1.080	1.267	1.132	0.863	0.585	0.430	0.267	0.999	1.140	0.973	0.786	0.840	0.589	0.682	1.515	1.172	1.098	0.755	0.622	0.697	0.573	0.993	0.423
E11	1.181	1.051	0.869	1.042	1.173	0.932	0.727	0.984	0.881	0.747	0.647	0.501	0.391	0.992	1.151	1.054	0.961	1.076	0.850	0.992	1.228	1.047	0.932	0.544	0.409	0.371	0.183	0.585	0.730
E12	0.768	0.670	0.480	0.709	0.878	0.680	0.482	0.789	0.755	0.825	0.910	0.823	0.788	1.115	1.263	1.244	1.236	1.380	1.196	1.353	0.993	1.039	0.903	0.603	0.555	0.403	0.373	0.272	1.116
E13	0.401	0.393	0.288	0.512	0.695	0.620	0.536	0.765	0.819	1.048	1.243	1.197	1.202	1.326	1.448	1.492	1.548	1.703	1.558	1.718	0.867	1.150	1.022	0.874	0.892	0.743	0.795	0.431	1.507
E14	0.362	0.510	0.584	0.646	0.763	0.836	0.861	0.967	1.077	1.381	1.631	1.610	1.636	1.629	1.725	1.813	1.911	2.070	1.955	2.113	0.949	1.395	1.287	1.250	1.300	1.161	1.241	0.840	1.924

Supplementary Table 4.2.3

The number of fascicles of each type within 0.5mm of each electrode around the nerve (nerve 7)

Pig	Threshold	Electrodes	Fascicle Type L	P	LP	C	CP
7	0.5mm	E1	2.0	0.0	0.0	0.0	0.0
		E2	6.0	0.0	1.0	0.0	0.0
		E3	2.0	0.0	1.0	0.0	0.0
		E4	0.0	0.0	1.0	0.0	0.0
		E5	0.0	3.0	1.0	0.0	0.0
		E6	0.0	5.0	0.0	0.0	0.0
		E7	0.0	3.0	0.0	0.0	0.0
		E8	0.0	3.0	0.0	0.0	1.0
		E9	0.0	2.0	0.0	0.0	1.0
		E10	0.0	2.0	0.0	0.0	1.0
		E11	0.0	1.0	3.0	0.0	0.0
		E12	2.0	0.0	2.0	1.0	0.0
		E13	3.0	0.0	0.0	1.0	0.0
		E14	1.0	0.0	0.0	0.0	0.0

Supplementary Table 4.2.4

The number of fascicles of each type within 0.5mm of each electrode around the nerve in % (nerve 7)

Pig	Threshold	Electrodes	Fascicle Type (%) L	P	LP	C	CP
7	0.5mm	E1	22.2	0.0	0.0	0.0	0.0
		E2	66.7	0.0	14.3	0.0	0.0
		E3	22.2	0.0	14.3	0.0	0.0
		E4	0.0	0.0	14.3	0.0	0.0
		E5	0.0	27.3	14.3	0.0	0.0
		E6	0.0	45.5	0.0	0.0	0.0
		E7	0.0	27.3	0.0	0.0	0.0
		E8	0.0	27.3	0.0	0.0	100.0
		E9	0.0	18.2	0.0	0.0	100.0
		E10	0.0	18.2	0.0	0.0	100.0
		E11	0.0	9.1	42.9	0.0	0.0
		E12	22.2	0.0	28.6	100.0	0.0
		E13	33.3	0.0	0.0	100.0	0.0
		E14	11.1	0.0	0.0	0.0	0.0

Supplementary Table 4.2.5

The number of fascicles of each type within 0.5mm of each electrode around the nerve reordered with cardiac at the top (to correspond with sVNS data) (nerve 7)

	Fascicle Type			
**Electrodes**	**'L'**	**'P'**	**'C'**	**CP'**
**E12**	2	0	1	0
**E13**	3	0	1	0
**E14**	1	0	0	0
**E1**	2	0	0	0
**E2**	6	0	0	0
**E3**	2	0	0	0
**E4**	0	0	0	0
**E5**	0	3	0	0
**E6**	0	5	0	0
**E7**	0	3	0	0
**E8**	0	3	0	1
**E9**	0	2	0	1
**E10**	0	2	0	1
**E11**	0	1	0	0
**max**	6	5	1	1

Supplementary Table 4.2.6

Supplementary Table 4.2.5 values normalized to 1 (yellow blocks) (nerve 7)

		Fascicle Type			
**Angle of Electrode**	**Electrodes**	**L**	**P**	**C**	**CP**
25.71429	1	0.333333	0	1	0
51.42857	2	0.5	0	1	0
77.14286	3	0.166667	0	0	0
102.8571	4	0.333333	0	0	0
128.5714	5	1	0	0	0
154.2857	6	0.333333	0	0	0
180	7	0	0	0	0
205.7143	8	0	0.6	0	0
231.4286	9	0	1	0	0
257.1429	10	0	0.6	0	0
282.8571	11	0	0.6	0	1
308.5714	12	0	0.4	0	1
334.2857	13	0	0.4	0	1
360	14	0	0.2	0	0

Nerve 8:

Supplementary Table 4.3.1

**Perimeter (mm)**	6.061385
**Area (mm2)**	2.28429

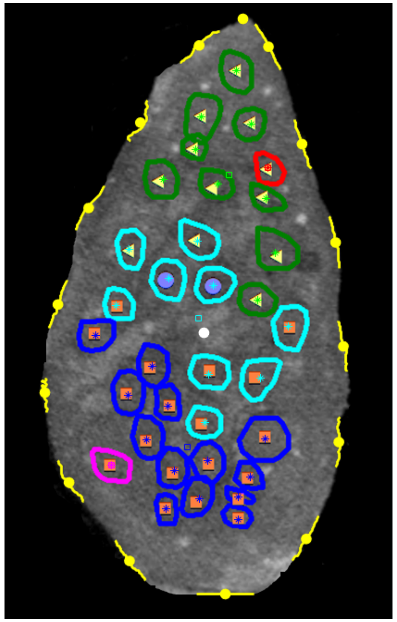

Supplementary Figure 4.3.1
Traced and labeled cross section of nerve 8

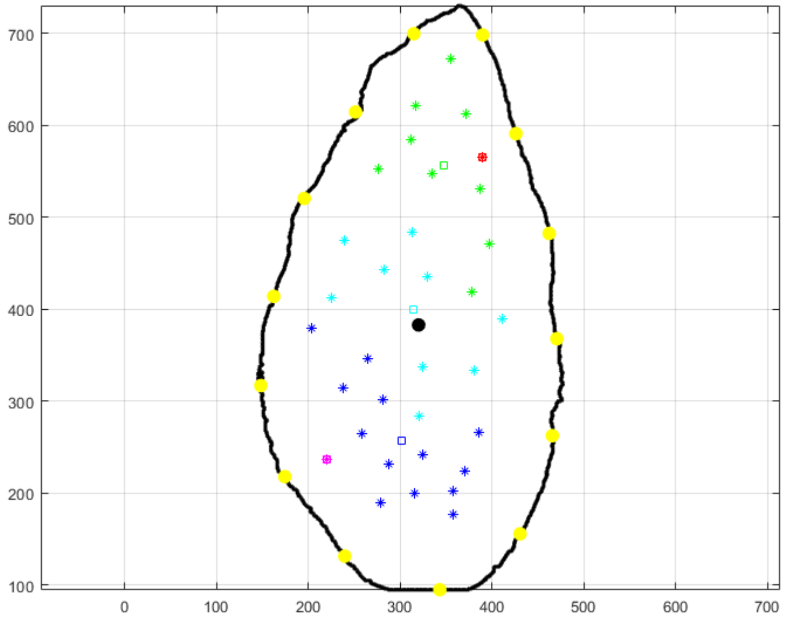

Supplementary Figure 4.3.2
Traced cross section mapped to determine co-ordinates and distance of fascicle centers of mass from the 14 electrodes for nerve 8

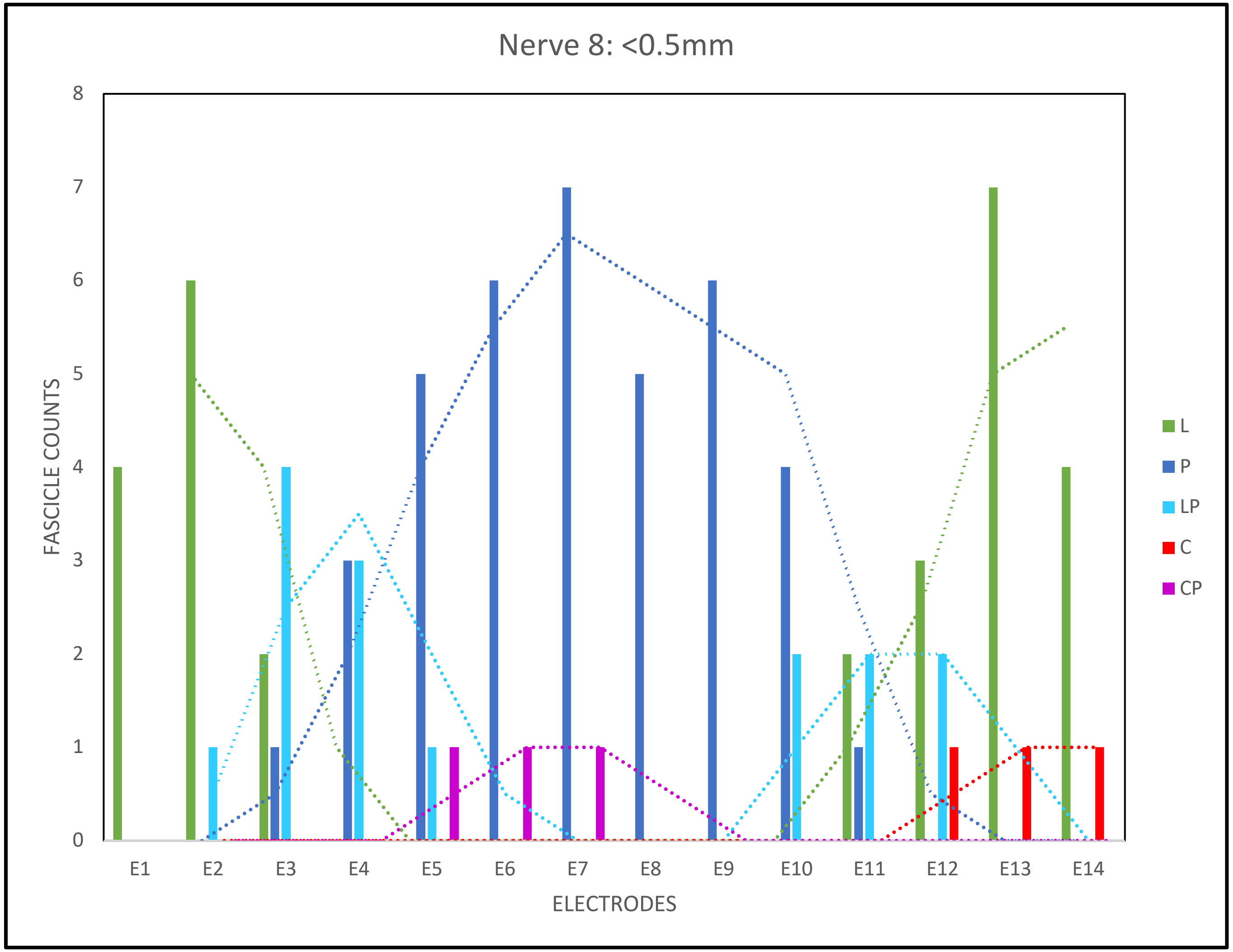

Supplementary Figure 4.3.3

A graph of the number and type of fascicles less than 0.5mm away from each electrode for nerve 8

Supplementary Table 4.3.2

The distance (mm) of each fascicle (and its type) within the cross section from each electrode around the circumference of the nerve (nerve 8)

Fascicle Progressive	1	2	3	4	5	6	7	8	9	10	11	12	13	14	15	16	17
Fascicle Type - Num	1	1	1	1	1	1	1	1	1	2	2	2	2	2	2	2	2
Fascicle Type - Char	'L'	'L'	'L'	'L'	'L'	'L'	'L'	'L'	'L'	'P'	'P'	'P'	'P'	'P'	'P'	'P'	'P'
E1	0.172	0.277	0.364	0.404	0.538	0.542	0.646	0.856	1.013	1.192	1.258	1.405	1.384	1.543	1.652	1.802	1.759
E2	0.417	0.232	0.421	0.235	0.237	0.379	0.561	0.720	0.820	0.843	0.947	1.105	1.059	1.231	1.357	1.501	1.477
E3	0.775	0.555	0.699	0.466	0.305	0.500	0.676	0.731	0.736	0.495	0.660	0.826	0.742	0.926	1.070	1.204	1.204
E4	1.133	0.909	1.015	0.798	0.629	0.767	0.892	0.850	0.760	0.187	0.431	0.575	0.442	0.624	0.782	0.893	0.926
E5	1.446	1.224	1.304	1.104	0.942	1.043	1.129	1.030	0.885	0.294	0.421	0.471	0.317	0.428	0.578	0.645	0.718
E6	1.720	1.505	1.553	1.379	1.229	1.289	1.332	1.187	1.008	0.579	0.550	0.478	0.406	0.338	0.403	0.383	0.500
E7	1.945	1.744	1.757	1.616	1.486	1.501	1.499	1.317	1.123	0.884	0.760	0.618	0.642	0.474	0.390	0.246	0.359
E8	2.031	1.854	1.826	1.728	1.626	1.591	1.542	1.337	1.147	1.118	0.926	0.761	0.855	0.669	0.516	0.400	0.382
E9	1.836	1.686	1.623	1.568	1.497	1.417	1.330	1.116	0.945	1.124	0.890	0.736	0.877	0.718	0.567	0.546	0.435
E10	1.493	1.366	1.278	1.259	1.218	1.102	0.985	0.773	0.631	1.013	0.768	0.664	0.821	0.731	0.635	0.706	0.574
E11	1.145	1.042	0.931	0.948	0.942	0.790	0.645	0.445	0.372	0.943	0.731	0.706	0.840	0.831	0.803	0.923	0.806
E12	0.766	0.706	0.560	0.643	0.699	0.502	0.315	0.233	0.371	0.981	0.847	0.900	0.987	1.050	1.077	1.218	1.121
E13	0.379	0.397	0.208	0.405	0.545	0.355	0.252	0.434	0.628	1.080	1.034	1.138	1.178	1.291	1.357	1.506	1.431
E14	0.153	0.373	0.307	0.485	0.652	0.566	0.589	0.801	0.984	1.299	1.316	1.446	1.454	1.594	1.683	1.834	1.773

Fascicle Progressive	18	19	20	21	22	23	24	25	26	27	28	29	30	31	32	33
Fascicle Type - Num	2	2	2	2	2	3	3	3	3	3	3	3	3	3	4	5
Fascicle Type - Char	'P'	'P'	'P'	'P'	'P'	'LP'	'LP'	'LP'	'LP'	'LP'	'LP'	'LP'	'LP'	'LP'	'C'	CP'
E1	1.611	1.547	1.686	1.758	1.846	0.759	0.835	0.911	1.057	0.933	1.142	1.312	1.277	1.463	0.541	1.662
E2	1.336	1.316	1.437	1.499	1.585	0.509	0.493	0.614	0.716	0.690	0.970	1.091	1.011	1.189	0.515	1.334
E3	1.080	1.119	1.211	1.257	1.336	0.434	0.222	0.409	0.393	0.561	0.887	0.929	0.791	0.943	0.699	1.002
E4	0.832	0.945	0.991	1.014	1.080	0.585	0.345	0.434	0.221	0.595	0.880	0.821	0.634	0.723	0.959	0.656
E5	0.673	0.856	0.847	0.839	0.885	0.826	0.641	0.647	0.433	0.763	0.960	0.822	0.627	0.620	1.217	0.379
E6	0.534	0.764	0.690	0.646	0.659	1.056	0.933	0.878	0.709	0.940	1.030	0.833	0.676	0.567	1.437	0.175
E7	0.490	0.700	0.563	0.482	0.444	1.267	1.209	1.107	0.992	1.115	1.092	0.867	0.784	0.609	1.614	0.378
E8	0.522	0.621	0.464	0.380	0.293	1.373	1.387	1.244	1.194	1.198	1.066	0.848	0.854	0.670	1.662	0.661
E9	0.482	0.418	0.321	0.305	0.269	1.227	1.310	1.138	1.159	1.045	0.827	0.648	0.738	0.593	1.448	0.794
E10	0.504	0.280	0.363	0.437	0.486	0.947	1.093	0.906	0.999	0.772	0.488	0.388	0.560	0.514	1.098	0.869
E11	0.680	0.466	0.618	0.708	0.782	0.689	0.898	0.714	0.879	0.548	0.223	0.338	0.524	0.604	0.750	0.995
E12	0.976	0.808	0.966	1.054	1.137	0.525	0.786	0.649	0.870	0.494	0.372	0.599	0.704	0.857	0.388	1.214
E13	1.280	1.153	1.307	1.391	1.477	0.547	0.774	0.726	0.945	0.644	0.709	0.922	0.962	1.142	0.159	1.441
E14	1.623	1.522	1.671	1.751	1.838	0.801	0.948	0.975	1.159	0.950	1.088	1.286	1.292	1.478	0.469	1.730

Supplementary Table 4.3.3

The number of fascicles of each type within 0.5mm of each electrode around the nerve (nerve 8)

Pig	Threshold	Electrodes	Fascicle Type L	P	LP	C	CP
8	0.5mm	E1	4.0	0.0	0.0	0.0	0.0
		E2	6.0	0.0	1.0	0.0	0.0
		E3	2.0	1.0	4.0	0.0	0.0
		E4	0.0	3.0	3.0	0.0	0.0
		E5	0.0	5.0	1.0	0.0	1.0
		E6	0.0	6.0	0.0	0.0	1.0
		E7	0.0	7.0	0.0	0.0	1.0
		E8	0.0	5.0	0.0	0.0	0.0
		E9	0.0	6.0	0.0	0.0	0.0
		E10	0.0	4.0	2.0	0.0	0.0
		E11	2.0	1.0	2.0	0.0	0.0
		E12	3.0	0.0	2.0	1.0	0.0
		E13	7.0	0.0	0.0	1.0	0.0
		E14	4.0	0.0	0.0	1.0	0.0

Supplementary Table 4.3.4

The number of fascicles of each type within 0.5mm of each electrode around the nerve in % (nerve 8)

Pig	Threshold	Electrodes	Fascicle Type (%) L	P	LP	C	CP
8	0.5mm	E1	44.4	0.0	0.0	0.0	0.0
		E2	66.7	0.0	14.3	0.0	0.0
		E3	22.2	9.1	57.1	0.0	0.0
		E4	0.0	27.3	42.9	0.0	0.0
		E5	0.0	45.5	14.3	0.0	100.0
		E6	0.0	54.5	0.0	0.0	100.0
		E7	0.0	63.6	0.0	0.0	100.0
		E8	0.0	45.5	0.0	0.0	0.0
		E9	0.0	54.5	0.0	0.0	0.0
		E10	0.0	36.4	28.6	0.0	0.0
		E11	22.2	9.1	28.6	0.0	0.0
		E12	33.3	0.0	28.6	100.0	0.0
		E13	77.8	0.0	0.0	100.0	0.0
		E14	44.4	0.0	0.0	100.0	0.0

Supplementary Table 4.3.5

The number of fascicles of each type within 0.5mm of each electrode around the nerve reordered with cardiac at the top (to correspond with sVNS data) (nerve 8)

	Fascicle Type			
**Electrodes**	**'L'**	**'P'**	**'C'**	**CP'**
**E12**	3	0	1	0
**E13**	7	0	1	0
**E14**	4	0	1	0
**E1**	4	0	0	0
**E2**	6	0	0	0
**E3**	2	1	0	0
**E4**	0	3	0	0
**E5**	0	5	0	1
**E6**	0	6	0	1
**E7**	0	7	0	1
**E8**	0	5	0	0
**E9**	0	6	0	0
**E10**	0	4	0	0
**E11**	2	1	0	0
**max**	7	7	1	1

Supplementary Table 4.3.6

Supplementary Table 4.3.5 values normalized to 1 (yellow blocks) (nerve 8)

		Fascicle Type			
**Angle of Electrode**	**Electrodes**	**'L'**	**'P'**	**'C'**	**CP'**
25.71429	1	0.428571	0	1	0
51.42857	2	1	0	1	0
77.14286	3	0.571429	0	1	0
102.8571	4	0.571429	0	0	0
128.5714	5	0.857143	0	0	0
154.2857	6	0.285714	0.142857	0	0
180	7	0	0.428571	0	0
205.7143	8	0	0.714286	0	1
231.4286	9	0	0.857143	0	1
257.1429	10	0	1	0	1
282.8571	11	0	0.714286	0	0
308.5714	12	0	0.857143	0	0
334.2857	13	0	0.571429	0	0
360	14	0.285714	0.142857	0	0

Nerve 9:

Supplementary Table 4.4.1

**Perimeter (mm)**	7.562024
**Area (mm2)**	3.40284

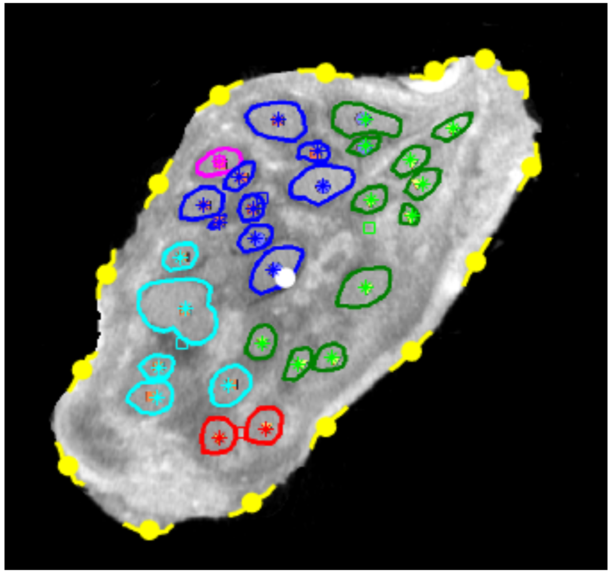

Supplementary Figure 4.4.1
Traced and labeled cross section of nerve 9

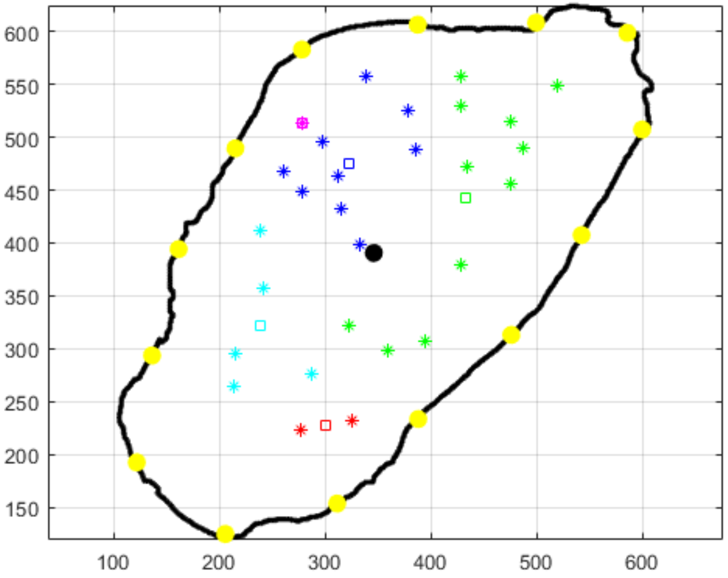

Supplementary Figure 4.4.2
Traced cross section mapped to determine co-ordinates and distance of fascicle centers of mass from the 14 electrodes for nerve 9

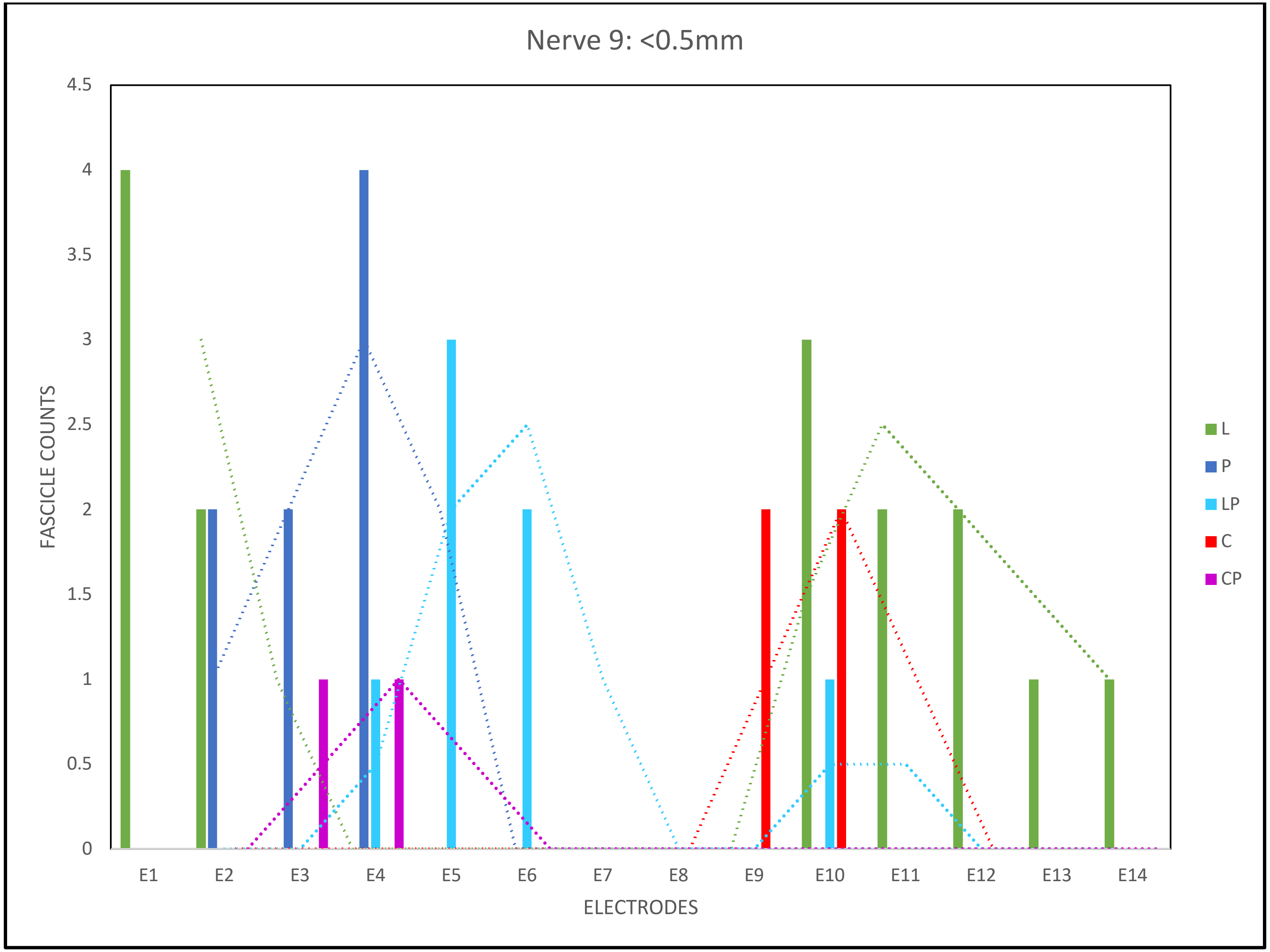

Supplementary Figure 4.4.3

A graph of the number and type of fascicles less than 0.5mm away from each electrode for nerve 9

Supplementary Table 4.4.2

The distance (mm) of each fascicle (and its type) within the cross section from each electrode around the circumference of the nerve (nerve 9)

Fascicle Progressive	1	2	3	4	5	6	7	8	9	10	11	12	13	14	15	16	17	18	19	20	21	22	23	24	25	26	27	28
Fascicle Type - Num	1	1	1	1	1	1	1	1	1	1	1	2	2	2	2	2	2	2	2	2	3	3	3	3	3	4	4	5
Fascicle Type - Char	'L'	'L'	'L'	'L'	'L'	'L'	'L'	'L'	'L'	'L'	'L'	'P'	'P'	'P'	'P'	'P'	'P'	'P'	'P'	'P'	'LP'	'LP'	'LP'	'LP'	'LP'	'C'	'C'	CP'
E1	0.279	0.382	0.464	0.422	0.520	0.674	0.662	1.046	1.474	1.487	1.397	0.738	0.641	0.727	1.010	1.213	1.197	1.035	1.117	1.170	1.431	1.572	1.722	1.849	1.957	1.815	1.947	1.055
E2	0.634	0.282	0.384	0.554	0.670	0.764	0.622	1.007	1.277	1.351	1.310	0.303	0.358	0.518	0.622	0.824	0.843	0.705	0.826	0.938	1.073	1.260	1.507	1.554	1.678	1.661	1.746	0.630
E3	1.070	0.671	0.702	0.912	1.001	1.029	0.838	1.107	1.158	1.292	1.310	0.290	0.510	0.626	0.390	0.512	0.590	0.541	0.680	0.840	0.768	0.996	1.339	1.285	1.420	1.549	1.575	0.306
E4	1.359	0.982	0.953	1.144	1.191	1.152	0.962	1.052	0.870	1.045	1.119	0.618	0.735	0.744	0.365	0.224	0.330	0.439	0.505	0.651	0.354	0.588	0.982	0.847	0.982	1.225	1.197	0.295
E5	1.708	1.372	1.312	1.472	1.486	1.404	1.242	1.174	0.772	0.961	1.088	1.055	1.112	1.062	0.746	0.541	0.563	0.727	0.693	0.752	0.345	0.388	0.753	0.490	0.609	1.009	0.905	0.729
E6	2.015	1.724	1.644	1.771	1.760	1.649	1.520	1.337	0.823	0.976	1.130	1.455	1.468	1.382	1.134	0.935	0.917	1.072	0.990	0.977	0.683	0.542	0.665	0.346	0.360	0.870	0.690	1.143
E7	2.336	2.086	1.994	2.093	2.062	1.932	1.834	1.576	1.044	1.139	1.293	1.857	1.840	1.732	1.536	1.347	1.310	1.451	1.347	1.293	1.087	0.895	0.813	0.610	0.510	0.907	0.693	1.560
E8	2.307	2.129	2.021	2.075	2.018	1.871	1.819	1.484	1.002	1.016	1.147	1.979	1.908	1.772	1.673	1.516	1.449	1.555	1.427	1.323	1.264	1.032	0.755	0.749	0.613	0.702	0.531	1.727
E9	1.952	1.838	1.721	1.735	1.660	1.506	1.492	1.116	0.737	0.669	0.762	1.768	1.651	1.498	1.498	1.388	1.296	1.357	1.217	1.077	1.174	0.943	0.549	0.751	0.649	0.347	0.338	1.578
E10	1.492	1.426	1.305	1.289	1.203	1.046	1.062	0.665	0.483	0.313	0.322	1.431	1.276	1.113	1.214	1.163	1.054	1.061	0.925	0.763	1.020	0.839	0.481	0.805	0.778	0.276	0.488	1.314
E11	1.045	1.087	0.967	0.883	0.775	0.625	0.719	0.358	0.676	0.516	0.360	1.224	1.020	0.863	1.117	1.157	1.049	0.975	0.876	0.730	1.128	1.043	0.843	1.145	1.171	0.751	0.957	1.231
E12	0.623	0.822	0.728	0.556	0.435	0.361	0.553	0.512	1.035	0.933	0.785	1.105	0.882	0.775	1.138	1.260	1.172	1.039	1.002	0.918	1.333	1.333	1.256	1.514	1.571	1.223	1.415	1.247
E13	0.393	0.779	0.754	0.548	0.500	0.587	0.742	0.933	1.462	1.394	1.257	1.163	0.970	0.944	1.322	1.494	1.433	1.274	1.290	1.261	1.638	1.697	1.700	1.922	1.998	1.703	1.883	1.409
E14	0.364	0.711	0.751	0.608	0.643	0.788	0.866	1.178	1.673	1.645	1.528	1.096	0.961	1.004	1.338	1.534	1.500	1.336	1.392	1.410	1.728	1.837	1.922	2.095	2.190	1.969	2.128	1.399

Supplementary Table 4.4.3

The number of fascicles of each type within 0.5mm of each electrode around the nerve (nerve 9)

Pig	Threshold	Electrodes	Fascicle Type				
			L	P	LP	C	CP
9	0.5mm	E1	4.0	0.0	0.0	0.0	0.0
		E2	2.0	2.0	0.0	0.0	0.0
		E3	0.0	2.0	0.0	0.0	1.0
		E4	0.0	4.0	1.0	0.0	1.0
		E5	0.0	0.0	3.0	0.0	0.0
		E6	0.0	0.0	2.0	0.0	0.0
		E7	0.0	0.0	0.0	0.0	0.0
		E8	0.0	0.0	0.0	0.0	0.0
		E9	0.0	0.0	0.0	2.0	0.0
		E10	3.0	0.0	1.0	2.0	0.0
		E11	2.0	0.0	0.0	0.0	0.0
		E12	2.0	0.0	0.0	0.0	0.0
		E13	1.0	0.0	0.0	0.0	0.0
		E14	1.0	0.0	0.0	0.0	0.0

Supplementary Table 4.4.4

The number of fascicles of each type within 0.5mm of each electrode around the nerve in % (nerve 9)

Pig	Threshold	Electrodes	Fascicle Type (%)				
			L	P	LP	C	CP
9	0.5mm	E1	36.4	0.0	0.0	0.0	0.0
		E2	18.2	22.2	0.0	0.0	0.0
		E3	0.0	22.2	0.0	0.0	100.0
		E4	0.0	44.4	20.0	0.0	100.0
		E5	0.0	0.0	60.0	0.0	0.0
		E6	0.0	0.0	40.0	0.0	0.0
		E7	0.0	0.0	0.0	0.0	0.0
		E8	0.0	0.0	0.0	0.0	0.0
		E9	0.0	0.0	0.0	100.0	0.0
		E10	27.3	0.0	20.0	100.0	0.0
		E11	18.2	0.0	0.0	0.0	0.0
		E12	18.2	0.0	0.0	0.0	0.0
		E13	9.1	0.0	0.0	0.0	0.0
		E14	9.1	0.0	0.0	0.0	0.0

Supplementary Table 4.4.5

The number of fascicles of each type within 0.5mm of each electrode around the nerve reordered with cardiac at the top (to correspond with sVNS data) (nerve 9)

	Fascicle Type			
**Electrodes**	**'L'**	**'P'**	**'C'**	**CP'**
**E9**	0	0	2	0
**E10**	3	0	2	0
**E11**	2	0	0	0
**E12**	2	0	0	0
**E13**	1	0	0	0
**E14**	1	0	0	0
**E1**	4	0	0	0
**E2**	2	2	0	0
**E3**	0	2	0	1
**E4**	0	4	0	1
**E5**	0	0	0	0
**E6**	0	0	0	0
**E7**	0	0	0	0
**E8**	0	0	0	0
**max**	4	4	2	1

Supplementary Table 4.4.6

Supplementary Table 4.4.5 values normalized to 1 (yellow blocks) (nerve 9)

		Fascicle Type			
**Angle of Electrode**	**Electrodes**	**'L'**	**'P'**	**'C'**	**CP'**
25.71429	1	0	0	1	0
51.42857	2	0.75	0	1	0
77.14286	3	0.5	0	0	0
102.8571	4	0.5	0	0	0
128.5714	5	0.25	0	0	0
154.2857	6	0.25	0	0	0
180	7	1	0	0	0
205.7143	8	0.5	0.5	0	0
231.4286	9	0	0.5	0	1
257.1429	10	0	1	0	1
282.8571	11	0	0	0	0
308.5714	12	0	0	0	0
334.2857	13	0	0	0	0
360	14	0	0	0	0

Nerve 10:

Supplementary Table 4.5.1

**Perimeter (mm)**	6.597412
**Area (mm2)**	2.63077

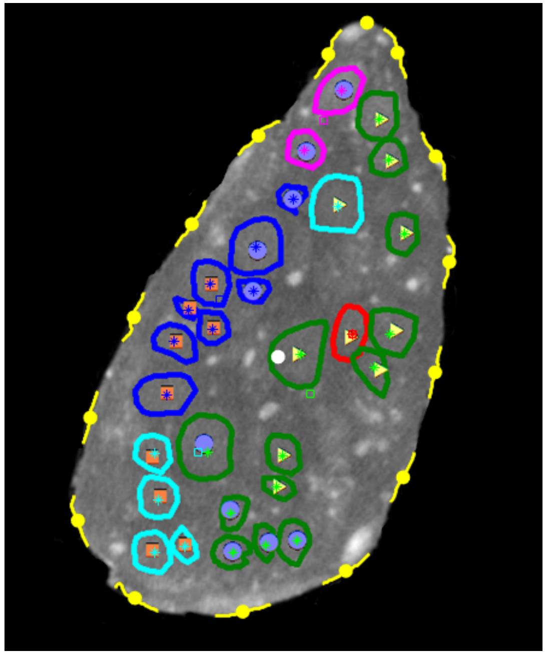

Supplementary Figure 4.5.1
Traced and labeled cross section of nerve 10

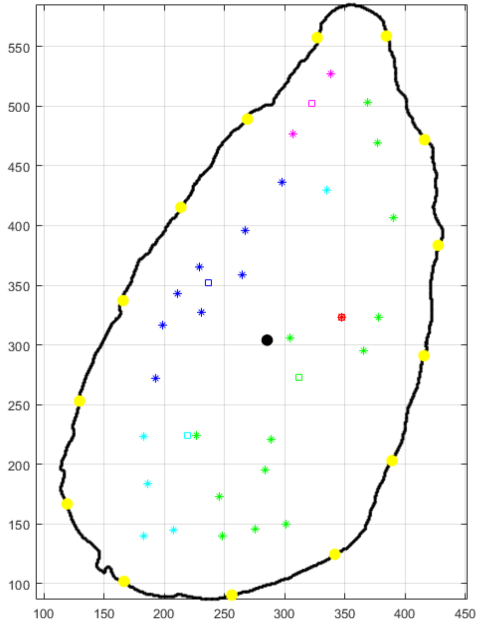

Supplementary Figure 4.5.2
Traced cross section mapped to determine co-ordinates and distance of fascicle centers of mass from the 14 electrodes for nerve 10

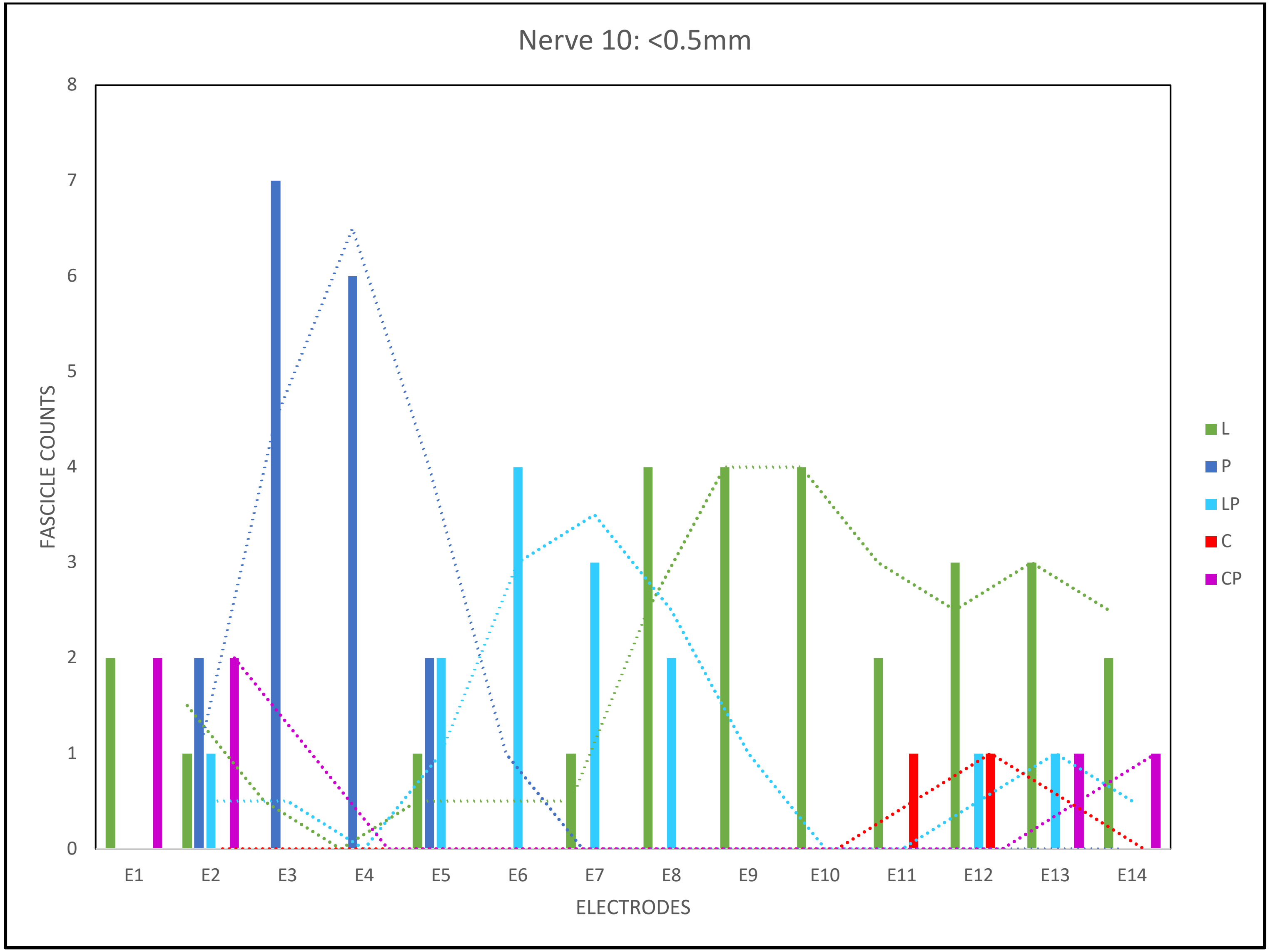

Supplementary Figure 4.5.3

A graph of the number and type of fascicles less than 0.5mm away from each electrode for nerve 10

Supplementary Table 4.5.2

The distance (mm) of each fascicle (and its type) within the cross section from each electrode around the circumference of the nerve (nerve 10)

Fascicle Progressive	1	2	3	4	5	6	7	8	9	10	11	12	13	14	15	16	17	18	19	20	21	22	23	24	25	26	27	28	29
Fascicle Type - Num	1	1	1	1	1	1	1	1	1	1	1	1	1	2	2	2	2	2	2	2	2	3	3	3	3	3	4	5	5
Fascicle Type - Char	'L'	'L'	'L'	'L'	'L'	'L'	'L'	'L'	'L'	'L'	'L'	'L'	'L'	'P'	'P'	'P'	'P'	'P'	'P'	'P'	'P'	'LP'	'LP'	'LP'	'LP'	'LP'	'C'	CP'	CP'
E1	0.327	0.482	0.774	1.138	1.258	1.197	1.606	1.731	1.651	1.863	2.015	1.966	1.936	0.592	0.817	0.986	1.024	1.156	1.184	1.291	1.495	0.606	1.726	1.893	2.094	2.035	1.116	0.393	0.154
E2	0.478	0.521	0.694	0.942	1.027	0.884	1.275	1.397	1.273	1.503	1.659	1.628	1.616	0.287	0.442	0.618	0.619	0.746	0.790	0.882	1.091	0.420	1.327	1.500	1.705	1.658	0.871	0.189	0.373
E3	0.845	0.815	0.837	0.893	0.916	0.672	0.988	1.096	0.909	1.159	1.315	1.309	1.324	0.411	0.268	0.361	0.249	0.343	0.426	0.471	0.686	0.579	0.923	1.104	1.313	1.282	0.768	0.530	0.792
E4	1.244	1.181	1.114	1.009	0.966	0.672	0.803	0.877	0.610	0.866	1.013	1.044	1.095	0.783	0.555	0.482	0.328	0.216	0.313	0.184	0.335	0.915	0.547	0.732	0.938	0.933	0.862	0.943	1.215
E5	1.641	1.557	1.434	1.223	1.133	0.864	0.771	0.782	0.482	0.669	0.776	0.856	0.948	1.179	0.940	0.815	0.710	0.575	0.595	0.447	0.314	1.285	0.290	0.422	0.592	0.633	1.083	1.355	1.632
E6	1.985	1.883	1.716	1.433	1.314	1.096	0.844	0.792	0.578	0.601	0.623	0.744	0.865	1.532	1.293	1.143	1.074	0.942	0.926	0.806	0.609	1.613	0.403	0.326	0.327	0.433	1.309	1.719	1.998
E7	2.130	2.006	1.793	1.451	1.313	1.167	0.810	0.711	0.646	0.506	0.426	0.555	0.677	1.702	1.474	1.305	1.282	1.163	1.110	1.032	0.816	1.749	0.580	0.400	0.197	0.284	1.353	1.899	2.173
E8	2.028	1.884	1.630	1.245	1.099	1.047	0.639	0.513	0.648	0.394	0.238	0.279	0.354	1.651	1.450	1.274	1.308	1.216	1.128	1.108	0.911	1.653	0.718	0.554	0.418	0.345	1.184	1.849	2.106
E9	1.799	1.642	1.359	0.957	0.816	0.878	0.521	0.431	0.719	0.508	0.449	0.331	0.227	1.491	1.335	1.169	1.259	1.206	1.093	1.135	0.992	1.448	0.885	0.789	0.755	0.640	0.941	1.679	1.908
E10	1.425	1.262	0.967	0.571	0.451	0.633	0.482	0.499	0.774	0.693	0.732	0.604	0.487	1.186	1.082	0.945	1.079	1.073	0.952	1.050	0.985	1.106	0.981	0.966	1.021	0.901	0.603	1.356	1.555
E11	1.029	0.863	0.563	0.234	0.240	0.533	0.685	0.772	0.948	0.979	1.069	0.957	0.861	0.885	0.862	0.783	0.951	1.000	0.891	1.034	1.059	0.762	1.148	1.200	1.314	1.203	0.358	1.021	1.178
E12	0.630	0.470	0.209	0.370	0.513	0.690	1.011	1.122	1.214	1.317	1.433	1.337	1.258	0.661	0.762	0.778	0.944	1.043	0.968	1.127	1.230	0.490	1.386	1.484	1.635	1.536	0.476	0.722	0.801
E13	0.267	0.185	0.332	0.729	0.873	0.949	1.334	1.455	1.478	1.631	1.764	1.684	1.621	0.585	0.793	0.894	1.021	1.148	1.114	1.264	1.420	0.432	1.617	1.747	1.923	1.837	0.778	0.517	0.452
E14	0.274	0.427	0.720	1.118	1.254	1.258	1.664	1.789	1.754	1.943	2.088	2.025	1.978	0.712	0.951	1.103	1.177	1.312	1.318	1.444	1.634	0.654	1.856	2.010	2.203	2.132	1.132	0.534	0.266

Supplementary Table 4.5.3

The number of fascicles of each type within 0.5mm of each electrode around the nerve (nerve 10)

				Fascicle Type				
**Year**	**Pig**	**Threshold**	**Electrodes**	**L**	**P**	**LP**	**C**	**CP**
2022	6	0.5mm	**E1**	2.0	0.0	0.0	0.0	2.0
			**E2**	1.0	2.0	1.0	0.0	2.0
			**E3**	0.0	7.0	0.0	0.0	0.0
			**E4**	0.0	6.0	0.0	0.0	0.0
			**E5**	1.0	2.0	2.0	0.0	0.0
			**E6**	0.0	0.0	4.0	0.0	0.0
			**E7**	1.0	0.0	3.0	0.0	0.0
			**E8**	4.0	0.0	2.0	0.0	0.0
			**E9**	4.0	0.0	0.0	0.0	0.0
			**E10**	4.0	0.0	0.0	0.0	0.0
			**E11**	2.0	0.0	0.0	1.0	0.0
			**E12**	3.0	0.0	1.0	1.0	0.0
			**E13**	3.0	0.0	1.0	0.0	1.0
			**E14**	2.0	0.0	0.0	0.0	1.0

Supplementary Table 4.5.4

The number of fascicles of each type within 0.5mm of each electrode around the nerve in % (nerve 10)

				Fascicle Type (%)				
**Year**	**Pig**	**Threshold**	**Electrodes**	**L**	**P**	**LP**	**C**	**CP**
2022	6	0.5mm	**E1**	15.4	0.0	0.0	0.0	100.0
			**E2**	7.7	25.0	20.0	0.0	100.0
			**E3**	0.0	87.5	0.0	0.0	0.0
			**E4**	0.0	75.0	0.0	0.0	0.0
			**E5**	7.7	25.0	40.0	0.0	0.0
			**E6**	0.0	0.0	80.0	0.0	0.0
			**E7**	7.7	0.0	60.0	0.0	0.0
			**E8**	30.8	0.0	40.0	0.0	0.0
			**E9**	30.8	0.0	0.0	0.0	0.0
			**E10**	30.8	0.0	0.0	0.0	0.0
			**E11**	15.4	0.0	0.0	100.0	0.0
			**E12**	23.1	0.0	20.0	100.0	0.0
			**E13**	23.1	0.0	20.0	0.0	50.0
			**E14**	15.4	0.0	0.0	0.0	50.0

Supplementary Table 4.5.5

The number of fascicles of each type within 0.5mm of each electrode around the nerve reordered with cardiac at the top (to correspond with sVNS data) (nerve 10)

	Fascicle Type			
**Electrodes**	**'L'**	**'P'**	**'C'**	**CP'**
**E11**	2	0	1	0
**E12**	3	0	1	0
**E13**	3	0	0	1
**E14**	2	0	0	1
**E1**	2	0	0	2
**E2**	1	2	0	2
**E3**	0	7	0	0
**E4**	0	6	0	0
**E5**	1	2	0	0
**E6**	0	0	0	0
**E7**	1	0	0	0
**E8**	4	0	0	0
**E9**	4	0	0	0
**E10**	4	0	0	0
**max**	4	7	1	2

Supplementary Table 4.5.6

Supplementary Table 4.5.5 values normalized to 1 (yellow blocks) (nerve 10)

		Fascicle Type			
**Angle of Electrode**	**Electrodes**	**L**	**P**	**C**	**CP**
25.71429	1	0.5	0	1	0
51.42857	2	0.75	0	1	0
77.14286	3	0.75	0	0	0.5
102.8571	4	0.5	0	0	0.5
128.5714	5	0.5	0	0	1
154.2857	6	0.25	0.285714	0	1
180	7	0	1	0	0
205.7143	8	0	0.857143	0	0
231.4286	9	0.25	0.285714	0	0
257.1429	10	0	0	0	0
282.8571	11	0.25	0	0	0
308.5714	12	1	0	0	0
334.2857	13	1	0	0	0
360	14	1	0	0	0
